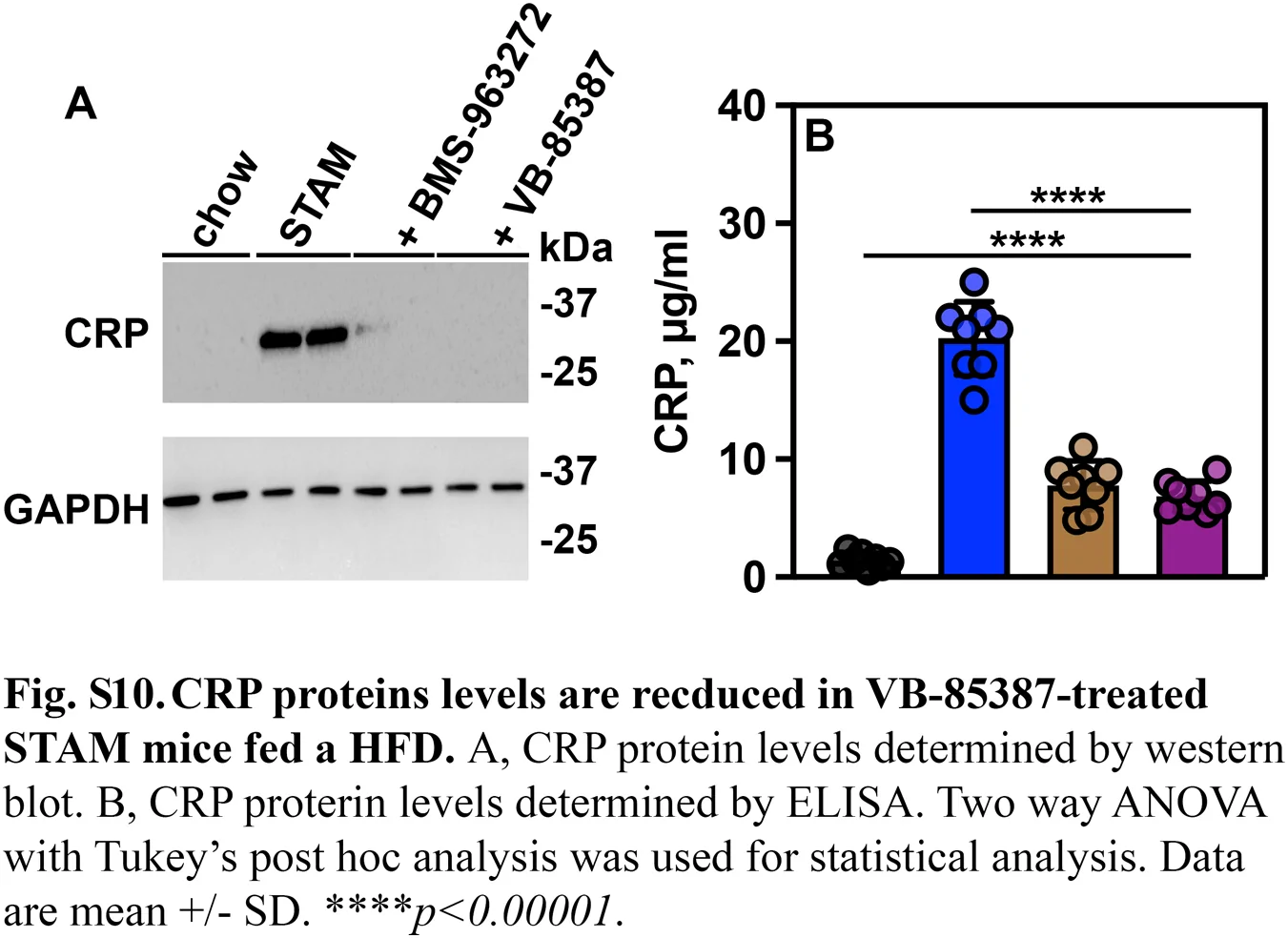# The monoacylglycerol acyltransferase 2 inhibitor VB-85387 reduces MASLD

**DOI:** 10.1016/j.jlr.2026.101137

**Published:** 2026-09-02

**Authors:** Christina Gallo-Ebert, Joshua Wayne, Alyssa Brown, Joseph T. Nickels

**Affiliations:** 1 The Institute of Metabolic Disorders, Genesis Biotechnology Group, Hamilton, NJ, USA; 2 Rutgers Center for Lipid Research, New Jersey Institute for Food, Nutrition, and Health, Rutgers University, New Brunswick, NJ, USA

**Keywords:** Liver, lipotoxicity, lipid, triglycerides, inflammation

## Abstract

Metabolic dysfunction-associated steatotic liver disease (MASLD) arises from the accumulation of triglycerides within the liver. MASLD can advance to metabolic dysfunction-associated steatohepatitis (MASH), cirrhosis, and hepatocellular carcinoma. Monoacylglycerol acyltransferase 2 (MOGAT2) is essential for triglyceride synthesis and plays a significant role in regulating lipid metabolism. Here, we demonstrate the ability of a new human MOGAT2 inhibitor, VB-85387, to inhibit the development of MASLD/MASH and further define its effects on the key metabolic pathways that progress MASH development. MASLD/MASH was induced using a low-methionine, choline-deficient diet or by streptozotocin treatment combined with high-fat diet feeding (STAM-HFD). VB-85387 significantly mitigated the severity of MASLD and reduced signs of MASH in mice subjected to these two distinct diets. VB-85387-treated mice exhibited decreased fibrosis, evidenced by reduced hepatic triglyceride concentrations, hydroxyproline levels, and collagen deposition. NAS scores were consistently lower in VB-85387-treated mice across both models. VB-85387-treated mice showed induced Pparα signaling and reduced Srebp transcription, demonstrating a likely role for VB-85387 in regulating lipogenesis and fatty acid β-oxidation. STAM-HFD treated mice showed lower NF-κBp65 activation, which was associated with lower tumor necrosis factor alpha expression. *Il-1β* and *Ifnβ* levels were also both reduced, suggesting VB-85387 can reduce proinflammatory pattern recognition receptor signaling. In addition, treatment suppressed interleukin 4/6-dependent Janus kinase activation. Overall, VB-85387 inhibited MASLD development by reducing liver triglyceride levels, fibrosis, and meta-inflammatory signaling. VB-85387 was as effective or superior to the MOGAT2 inhibitor phase I clinical trial drug BMS-963272 in reducing MASLD and fibrosis. VB-85387 has considerable potential for developing therapeutics targeting MASLD/MASH.

## Graphical abstract

**Figure:**
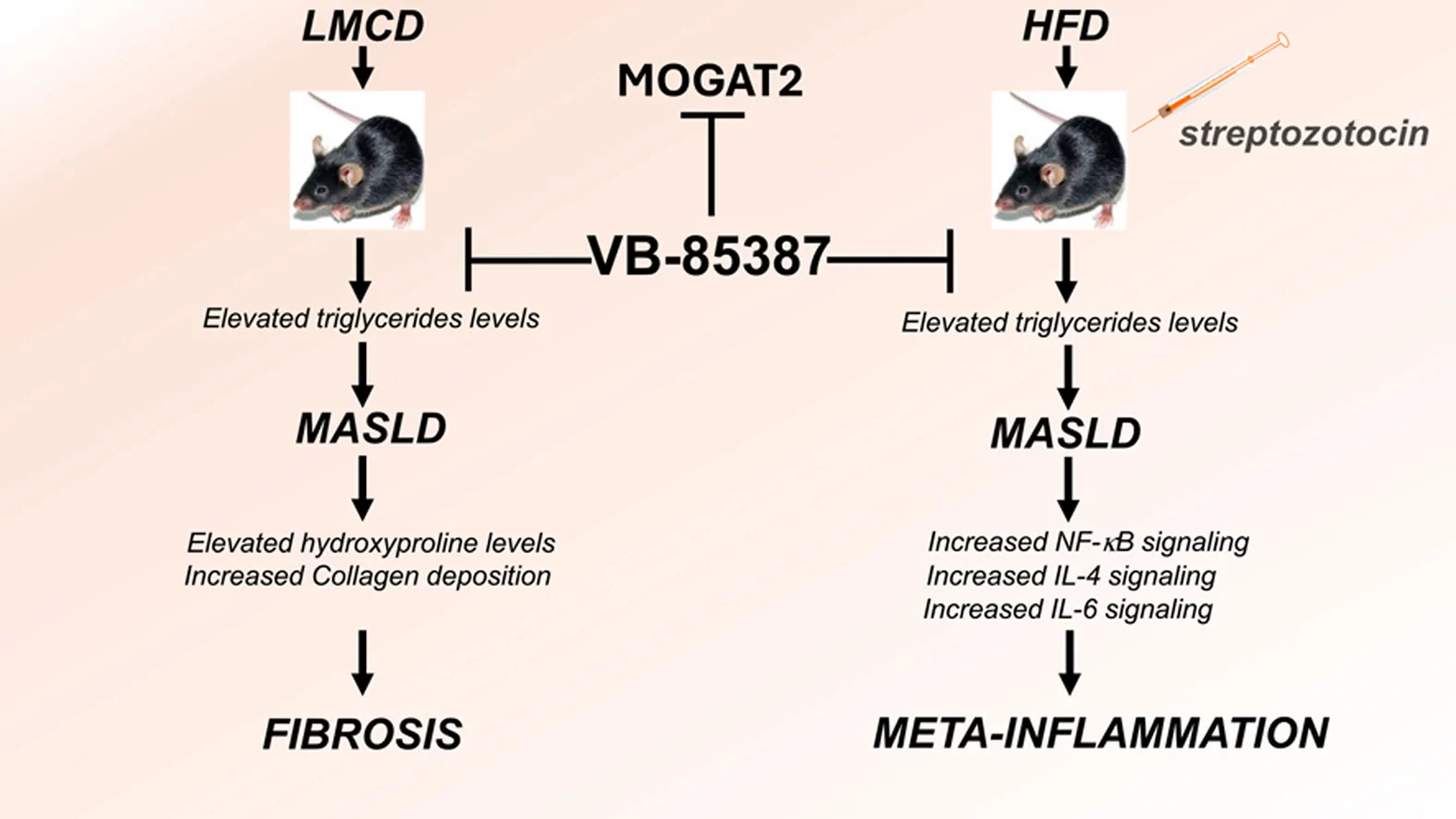

It is estimated that in the United States alone, 40% of the population has metabolic dysfunction-associated steatotic liver disease (MASLD), and of those individuals, 20% have progressed to metabolic dysfunction-associated steatohepatitis (MASH) (1, 2). If MASH is not treated, it often progresses to cirrhosis and hepatocellular carcinoma (3, 4). Recently, the thyroid hormone receptor-β agonist, Rezdiffra (5), and the GLP-1 receptor agonist, Wegovy (6), have been approved to treat MASH. Important guidelines have been published for using these therapies (6, 7) since both drugs commonly cause side effects such as diarrhea and vomiting (8, 9). Furthermore, more serious issues have also been identified including muscle loss and anorexia, and in rare occasions, liver injury (10), pancreatitis, and gall bladder disease (6). The search for new drugs to treat MASLD/MASH continues, with clinical trials exploring treatments such as the GLP-1/GIP dual receptor agonist survodutide (11), the FGF21 analogs efruxifermin and pegozafermin (12, 13, 14), and pan-PPAR agonists (lanifibranor, saroglitazar, and chiglitazar) (15, 16, 17), attempting to broaden available therapeutic options (18). Recently attention has turned to targeting human monoacylglycerol acyltransferase 2 (MOGAT2) (19).

MOGAT1, MOGAT2, and MOGAT3 are human MOGATs and members of the diacylglycerol acyltransferase 2 family (20, 21, 22, 23, 24). MOGAT2 plays a key role in catalyzing the synthesis of diacylglycerides, which serve as precursors for triacylglycerides production (21, 22, 25). Its enzymatic activity is predominantly observed in hepatic tissue and the duodenum (21, 22, 26, 27, 28). In the intestine, MOGAT2 is essential for the resynthesis of dietary triglycerides, and facilitates their transport from the lumen, across the intestinal epithelium to the liver in chylomicrons (27). Therefore, MOGAT2 is vital for maintaining appropriate triglycerides levels and overall lipid metabolism.

Mice that lack *mMogat2* have reduced liver triglycerides levels and are protected from hepatic steatosis when fed a high-fat diet (HFD) (29). They exhibit delayed triglyceride absorption in the small intestine, which may also help prevent fatty liver (30). Introducing human *hMOGAT2* into the intestine of these mice restores the development of hepatic steatosis (31). When *mMogat2* is ablated in adult mice, they also show decreased hepatic steatosis (32), suggesting that drugs which inhibit MOGAT2 may be useful during active disease progression. Indeed, several MOGAT2 inhibitors have been found to reduce MASLD in mouse models of fatty liver disease (19, 33, 34, 35, 36). Thus, eliminating MOGAT2 expression or inhibiting it pharmacologically significantly slows MASLD progression.

Accumulation of fat in the liver leads to meta-inflammation due to proinflammatory cytokine release (37). In addition, dysfunctional adipocytes produce adipokines that draw macrophages into the liver (38). Resident hepatic Kupffer cells also secrete cytokines such as TGF-β1 (39) and initiate *TΝFα* expression via NF-κB signaling (40); chemokines that bring in more monocytes and immune cells are also secreted (39).

These processes trigger several proinflammatory pathways, including interleukin-4 (*IL-4*)/interleukin-6 (*IL-6*)-induced Janus kinase 1 (JAK1)-signal transducer and activator of transcription 3/6 (STAT3/6) signaling (41), the IL-17-dependent p38 MAPK cascade (42), and IL-22 activity (43), and these in turn activate multiple signaling routes such as JAK-STAT, JNK, ERK1/2, and PI3K/AKT (37). The activation of these pathways contributes to the lipid-driven meta-inflammation seen in MASLD and facilitates its progression to MASH (37).

The present study used two distinct, complementary diet induced models of hepatic steatosis to demonstrate that the novel MOGAT2 inhibitor VB-85387 significantly reduces MASLD. In both models, VB-85387 treatment lowered hepatic triglyceride levels and reduced key histological and biochemical markers of steatosis and fibrosis compared with untreated disease controls. In the STAM-HFD model, VB-85387 reduced *IL-4*/*IL-6* expression and JAK1 activation, suggesting that MOGAT2 inhibition suppresses lipid-driven meta-inflammation. Overall, these findings indicate that VB-85387 mitigates MASLD and fibrosis thereby attenuating MASH by reducing hepatic triglyceride accumulation and reducing lipid-dependent proinflammatory signaling.

## Materials and methods

### Miscellaneous reagents

Miscellaneous reagents were purchased from Millipore Sigma (Burlington, MA). H&E stain was from Abcam (Waltham, MA; #ab245880). Masson’s trichrome C reagent was from Millipore Sigma (Burlington, MA; #HF15). Western blot supplies were from Bio-Rad (Hercules, CA). ELISA reagents were from R&D Systems (Minneapolis, MN). Organic solvents were purchased from Thermo Fisher Scientific (Waltham, MA).

### Pharmacokinetics analysis

Single-dose pharmacokinetics of VB-85387 were evaluated in C57BL/6 mice. The animals received either an intravenous dose of 1 mg/kg or an oral dose of 10 mg/kg of compound. Plasma concentrations of VB-85387 were determined by LC-MS/MS, using optimized standard calibration curves and quality control samples. Plasma was collected at predetermined intervals over a 24-h period, with three mice sampled at each time point. Pharmacokinetic parameters were calculated from the average plasma concentration-time profiles using the standard noncompartmental method with PKSolver.

### Animal studies

Administration of an low-methionine, choline-deficient diet (LMCD) diet to mice is a recognized approach for investigating various aspects of MASLD and MASH that relate to human disease etiologies. This model enables the study of progressive lipid accumulation resulting in fatty liver (44), hepatic inflammation associated with heightened oxidative stress (45), the onset of liver fibrosis (44), and progression towards hepatocellular carcinoma (46).

### Sixteen weeks LMCD diet study

Six-week-old male and female C57BL/6T mice (Taconic Biosciences, Germantown, NY) were housed individually under a 12-h light/dark cycle with ad libitum access to food and water (n = 8). Mice were acclimated for two weeks on a standard chow (Purina Picochow 5053, Lab Diets). 8-week-old mice were fed a LMCD for 16 weeks; VB-85387 or comparator was incorporated into the diet at 30 mg/kg beginning at week 8 and continued for 8 weeks (supplemental Fig. S1) (n = 8). The human MOGAT2 inhibitor, BMS-963272, served as a comparator compound (19, 47).

### 24 weeks LMCD diet study

8-week-old C57BL/6T mice were fed an LMCD for 24 weeks; VB-85387 or comparator were incorporated into the diet at 30 mg/kg beginning at week 8 and continued for 16 weeks (supplemental Fig. S2) (n = 8).

Taste aversion assessments indicated no variation in food intake among the groups. No evidence of steatorrhea was observed in mice administered either compound.

### STAM-HFD study

Postnatal day-2 pups received a subcutaneous injection of streptozotocin (200 μg) and were placed on an HFD at 4 weeks of age (supplemental Fig. S3). VB-85387 or comparator were incorporated into the diet beginning at 6 weeks of age and continued for 16 weeks.

All animal procedures in this study were conducted in accordance with the guidelines set forth by the Association for Assessment and Accreditation of Laboratory Animal Care. The Invivotek Institutional Animal Care and Use Committee approved all protocols (AUP protocol #1583). All experimental procedures adhered to the standards described in the "Institutional Animal Care and Use Committee Handbook".

### Oral lipid tolerance test

Eight-week-old female C57BL/6 mice were fasted overnight (n = 3; each time point). Mice were given 5 ml/kg olive oil by oral gavage. Triglycerides levels were determined at 0, 1, 2, 4, and 6 h after administration using a Cardiochek meter (PTS Diagnostics, Whitestown, IN). Two-way Anova with Tukey’s post hoc was used for calculations.

### Lipid extraction from mouse liver

100 mg of liver tissue was homogenized on ice using a mechanical tissue homogenizer. Tissue homogenate was extracted with 18 volumes of hexane:2-propanol (3:2). Samples were vortexed and subsequently centrifuged at 5,000 X *g*. The supernatant was transferred to a glass conical tube and subsequently washed with one-fifth volume of 0.9% NaCl. Samples were centrifuged at 5,000 X *g* and the aqueous phase was removed. The lower organic phase was dried down under nitrogen and resuspended in isopropyl alcohol until use.

### Lipid extraction from mouse small intestines

Duodenum samples (50 mg) were homogenized with 2 mg sodium sulfate. Chloroform: methanol (2:1) was added, and tissue samples were vortexed and incubated overnight at 4°C. Sodium chloride (0.7%) was added, and phase separation was allowed to proceed overnight at 4°C. The lower organic phase was collected, evaporated under nitrogen, and reconstituted in 2-propanol.

### Lipid extraction from mouse feces

Fecal samples were first lyophilized and then pulverized to a fine powder. Lipids were extracted with chloroform: methanol (2:1), vortexed, and centrifuged to separate the aqueous and organic phases. The organic phase was collected and dried under nitrogen. The recovered dried lipid fraction was weighed and normalized to total fecal mass.

### Lipid level determinations

Lipid levels were measured in serum collected by cardiac puncture, as well as in liver, duodenal intestine, epididymal adipose tissue, and fecal samples. Total cholesterol was quantified using an Abcam assay kit (Waltham, MA; #285242), and total triglycerides were measured using an Abcam kit (#65336). Total free fatty acid levels were determined using Abcam kit #ab65341.

### Apolipoprotein analysis

HDL, LDL, and VLDL concentrations were measured using the LipoPrint LDL system (LipoPrint LDL Subfraction kit #48–7002; Quantimetrix, Redondo Beach, CA). The Lipoprint results provide direct quantification of each particle size as a relative percentage. Absolute values for each particle type were calculated by applying the relative percentage obtained from the Lipoprint system to the total cholesterol concentration previously determined (relative percentage of total cholesterol multiplied by total cholesterol).

### Serum clinical chemistry analysis

Serum was collected from mice by cardiac puncture at the end of each study (n = 8). Serum samples were analyzed on an ACE Alera (Alfa Wasswerman) for liver enzyme levels according to the manufacturer’s instructions.

### Hydroxyproline assay

Hydroxyproline was measured as previously described (48). Liver tissues were suspended in autoclave-safe tubes in 100 ml 50 mM K_3_PO_4_ (pH 7.0), 5 mM EDTA, and 5 mM N-acetyl-L-cysteine (100 ml total). After adding 100 ml of 4M NaOH, samples were autoclaved at 120°C, 15 psi for 15 min, cooled, and adjusted to pH 7 with 4M HCl. Chloramine T solution was added, followed by incubation at room temperature for 20 min. Ehrlich’s solution was then added; samples were vortexed and incubated at 65°C for 20 min before stopping the reaction on ice. Hydroxyproline levels were determined as absorbance (550–565 nm) of a 20 μl sample, in 96-well plates.

### Histology and computer-aided image processing

Left liver lobes (n = 8) were stored for histology in 10% neutral buffered formalin, embedded in paraffin, sectioned at 5 μm, mounted, dehydrated with ethanol, and delipidated. Sections were stained with hematoxylin and eosin (H&E) or Masson’s trichrome C. Digital analysis was conducted employing a machine learning algorithm to quantify percentage lipid area, percentage fibrosis area, and immune cell density (Reveal Sciences, San Diego, CA). A certified veterinary pathologist carried out the histological staging. NASH activity score (NAS) was calculated by summing up the steatosis (0–3), inflammation (0–3), and hepatocyte ballooning (0–2), staging values (49). Fibrosis staging is shown separately. A NAS value of ≥5 was used as a cutoff for the presence of MASH (50).

### Quantifying size and number of adipocytes

Adipocyte size and quantity were assessed according to the methodology described (51). Adipose tissue histology samples underwent H&E staining. The area of individual adipocytes was quantified using ImageJ software. The distribution of adipocyte areas was used to calculate cell population numbers as represented by the area under the curve (AUC).

### Protein extraction

Tissues were dissected and cleaned of adhering fat and soft tissues. They were then washed in ice-cold PBS to remove blood, snap-frozen in liquid nitrogen, and stored at −80°C until further processing.

Total tissue lysates were prepared by homogenization of tissues in radioimmune precipitation buffer containing phosphatase and protease inhibitors, followed by the removal of tissue debris by centrifugation. Protein concentrations were determined using the Pierce™ BCA protein Assay Kit (Thermo Fisher Scientific, Waltham, MA).

### Western blotting

Tissue lysates were resuspended in protein sample buffer and incubated at 95°C for 10 min. Protein levels were measured with the Pierce™ BCA assay kit. Each panel shown in the results section reflects a separate Western blot run on individual SDS-PAGE gels. Densitometry was performed using Image J software for accurate protein quantification (mean ± SD) (n = 3).

25 μg of protein in sample buffer was loaded per well and resolved using 4%–20% SDS-PAGE gels, then transferred to nitrocellulose membranes. Membranes were blocked with TBST (Tris-buffered saline, 0.1% Tween 20) plus 10% nonfat dry milk for 1 h to overnight, then washed five times with TBST. After incubation with primary antibodies overnight in TBST, membranes were again washed thoroughly before incubation with secondary antibodies for 1–4 h. Following five additional TBST washes, membranes were incubated with chemiluminescent reagent and exposed for 2–5 min. GAPDH served as a loading control.

The antibody dilutions (TBST) used were as follows. Anti-pp65 monoclonal antibody (Cell Signaling (7F1) #3036; 1:1000); anti-p65 monoclonal antibody Cell Signaling (L8F6) #64921; 1:1500); anti-pJAK1^Tyr1022/1023^ monoclonal antibody (Cell Signaling (D7N4Z) #74129; 1:750) anti-JAK1 antibodies (Abcam [EPR24649-58] #ab324683; 1,200); anti pSTAT6^Tyr641^ (Santa Cruz [pY641.18] #sc-136019; 1:2000); anti-STAT6 antibodies (Abcam [YE361] #ab32520; 1:500); anti-C-reactive protein (CRP) antibodies (Santa Cruz [26D7] #sc-69770; 1:2500); anti-COL1A1 antibodies (Abcam, #ab21286; 1:750); anti-COL3A1 antibodies (Abcam [EPR17673], #ab184993; 1:1500); αSMA antibodies (Abcam, [1A4], #ab7817; 1:1000); anti-GAPDH antibodies (Abcam, [EPR16891] #ab181602; 1:5000).

### ELISA assays

ELISA assays were performed as per manufacturers' protocols. Briefly, controls and samples were added to wells and incubated for 2–4 h at room temperature or overnight at 4°C with gentle shaking. Wells were washed, then biotinylated antibodies were added, followed by HRP-streptavidin and a 1 h incubation. After another wash, substrate reagent was added and incubated in the dark for 30 min before stop solution. Plates were read at 450 nm immediately. pIκBα and total IκBα (Abcam, #ab279827); pJAK1 and total JAK1 (Fortis Life Sciences, #E172-003); *IL-6* (Abcam, #ab222503); *IL-4* (Abcam, #ab100710); CRP (Abcam, #ab222511); *Il-1β* (Abcam #ab197742); *Ifnβ* (Abcam #ab252363); COL1A1 (Abcam, #ab210579); COL3A1 (LS Bio, #LS-F25411); αSMA (LS Bio, #LS-F21849) kits were used for analysis.

### Apoptosis protein microarray analysis

Apoptosis signaling was assessed using the RayBiotech Mouse Apoptosis Signaling Pathway Phosphorylation Array C1 (#AAM-APOSIG-1-2; Peachtree Corners, GA) according to the manufacturer’s protocol. Membranes were blocked for 30 min at room temperature and then incubated overnight at 4°C with liver tissue lysates. After aspiration and repeated washing, membranes were incubated with the provided biotinylated antibody cocktail for 2 h at room temperature and washed again. HRP-streptavidin solution was added to each well, followed by a 2 h incubation and additional washes. Detection solution was then applied for 2 min, and membranes were exposed after 5 min in detection reagent. Data represent duplicate analyses of individual liver samples (n = 3).

### Cytokine protein microarray analysis

Cytokine protein levels were measured using the RayBiotech Mouse Cytokine Array GS1000 (#GSM-CAA-1000) according to the manufacturer’s instructions. The GS1000 microplate detects 80 cytokines. Membranes were blocked with the supplied buffer for 30 min and then incubated overnight at room temperature with sample lysates. Slides were washed three times with wash buffer 1 and twice with wash buffer 2. After incubation with the biotinylated antibody cocktail for 2 h at room temperature, slides were washed again. Streptavidin-conjugated fluor was then added, and slides were incubated for 2 h before final washes. Slides were scanned using a Typhoon 9500 scanner (GE HealthCare Technologies, Chicago, IL).

### RNA isolation

Total RNA was extracted from liver tissue using the RNeasy® Mini kit (Qiagen, Germantown MD) as per manufacturer’s protocol. Murine liver (50 mg) was homogenized in RLT buffer with a Bullet Blender® 24 Gold, followed by addition of 100% ethanol. Samples were loaded onto an RNeasy column, washed with RW1, treated with RNase-Free DNase, then washed with RW1 and RPE buffers. RNA was eluted in RNase-free water, quantified with a NanoDrop® ND-1000 spectrophotometer, and stored at −80°C. Reverse transcription was performed using the QuantiTect Reverse Transcription kit (Qiagen, Germantown, MD).

### Statistics

Data were analyzed using a two-way Anova analysis with Tukey’s post hoc analysis. Data are presented as mean ± SD.

## Results

### VB-85387 pharmacokinetic parameters

Our prior studies have shown that VB-85387 (5-((6-(5-methyl-2-(N-(thiophene-2-carbonyl)sulfamoyl)phenyl)pyridin-3-yl)methoxy)-N-(3-(trifluoromethyl)benzyl)picolinamide) (Fig. 1A) acts as a potent and selective inhibitor of both human and mouse MOGAT2 (52). It demonstrates nanomolar potency against human (IC_50_; 11.0 nM) and mouse MOGAT2 (IC_50_; 10.4 nM), exhibiting greater than 1,000-fold selectivity over related acyltransferases, including MOGAT1/3, diacylglycerol acyltransferase 1/2, ACAT1/2, and AWAT1/2. VB-85387 has IC_50_ values for MOGAT2 inhibition comparable to those of the phase I MOGAT2 comparator inhibitor (human IC_50_ = 7.1 nM; mouse IC_50_ = 18 nM) (47).

**Fig. 1:**
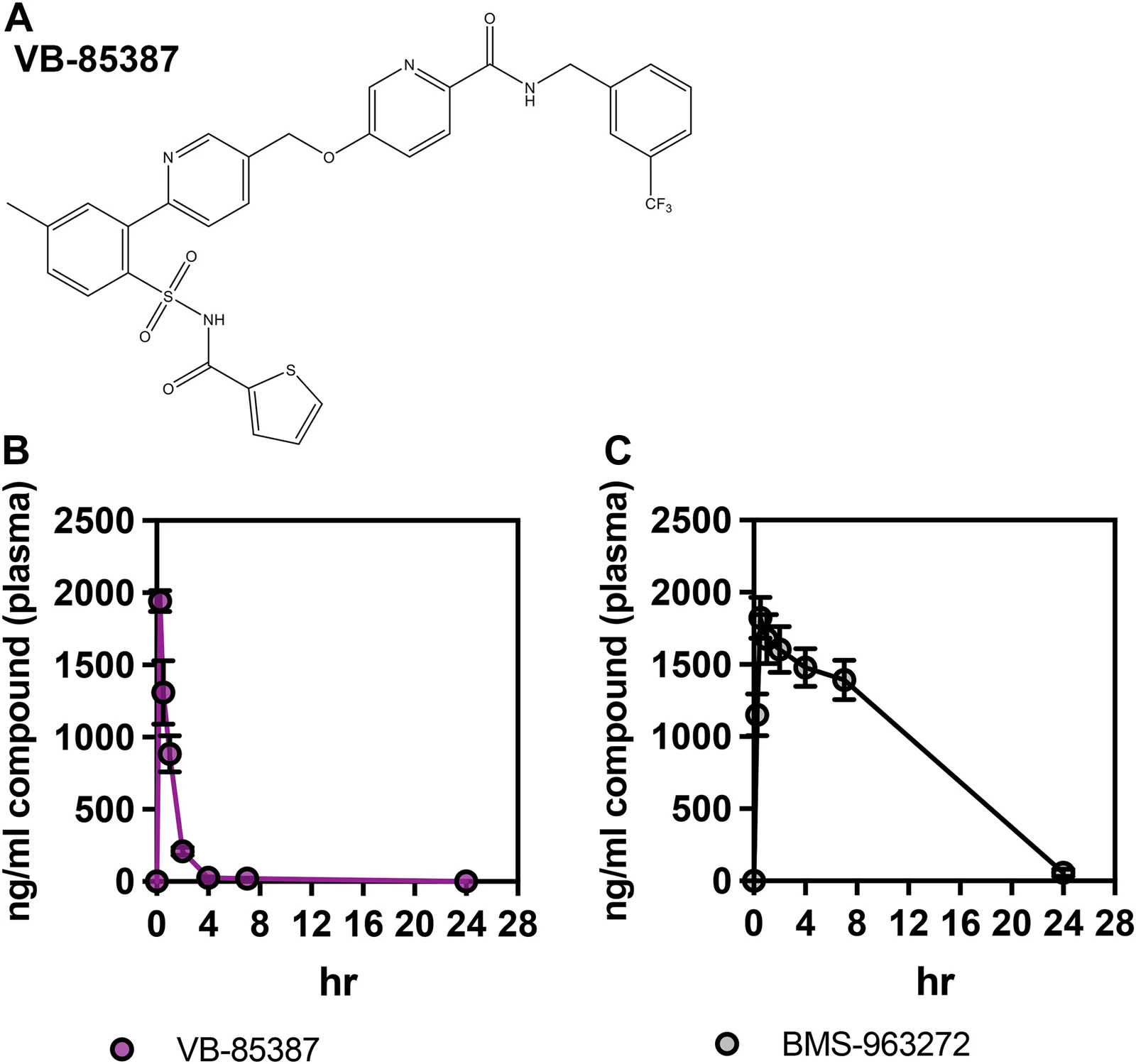
Chemical structure of VB-85387. A: Chemical structure of VB-85387 (5-((6-(5-methyl-2-(N-(thiophene-2-carbonyl)sulfamoyl)phenyl)pyridin-3-yl)methoxy)-N-(3-(trifluoromethyl)benzyl)picolinamide). B: Plasma concentration-time profile of intravenously administered VB-85387 at 1 mg/kg. C: Plasma-concentration time profile of intravenously administered comparator at 1 mg/kg.

Compound VB-85387 is a highly lipophilic small molecule (cLogP = 7.1) that is extensively protein-bound (>99.9% in both human and mouse plasma). The compound exhibits a favorable pharmacokinetic profile in mice, highlighted by exceptional oral bioavailability (F = 95%) and a volume of distribution of 9.9 L/kg (Table 1). Intravenous administration at 1 mg/kg revealed a high systemic clearance (67 ml/min/kg), contributing to a relatively short terminal half-life of 1.7 h (Fig. 1B), compared to that of comparator (4.9 h) (Fig. 1C). Despite this rapid clearance, oral dosing at 10 mg/kg yielded appreciable total systemic exposure (C_max_ = 2,018 ng/ml; AUC = 2,367 ng h/ml, T-half = 2.6 h), with the high volume of distribution and lipophilic character supporting the likelihood of favorable hepatic and intestinal partitioning relevant to in vivo evaluation in MASH disease models.

**Table 1:** PK parameters for VB-85387

VB-85387	IV	PO
Dose (mg/kg)	1	10
Cmax (ng/ml)	642	2,018
Tmax (hr)	0.083	0.25
AUC (0-t) (ng/ml∗hr)	245	2,367
AUC (0_inf_obs) (ng/ml∗hr)	249	2,367
T_1/2_ (hr)	1.7	2.6
CL (ml/min/kg)	67	-
Vz (L/kg)	9.9	-
F_abs_ %	-	95

### Male and female mice treated with VB-85387 show reduced development of MASLD and fibrosis in the LMCD model

Female mice have been shown to be resistant to diet-induced MASLD when fed a HFD or high-sugar Western diet (53, 54). However, the LMCD diet overcomes this protection and induces rapid hepatic steatosis and fibrosis in both sexes (55, 56). To determine whether female mice respond similarly to male mice under LMCD feeding conditions, male and female mice were fed the LMCD for 16 weeks (supplemental Fig. S1). Beginning at week 8, mice were treated with 30 mg/kg VB-85387 in comparison with 30 mg/kg comparator compound.

Histological analysis showed that both male and female mice fed the LMCD developed hepatic steatosis under these dietary conditions (Fig. 2, H&E). Treatment with VB-85387 markedly reduced the development of hepatic steatosis. Both male and female mice also showed early evidence of fibrosis, (Fig. 2). As expected, fibrosis was more pronounced in male mice than in female mice. VB-85387 reduced fibrosis in both male and female mice to a greater extent than comparator compound (Fig. 2, trichrome C).

**Fig. 2:**
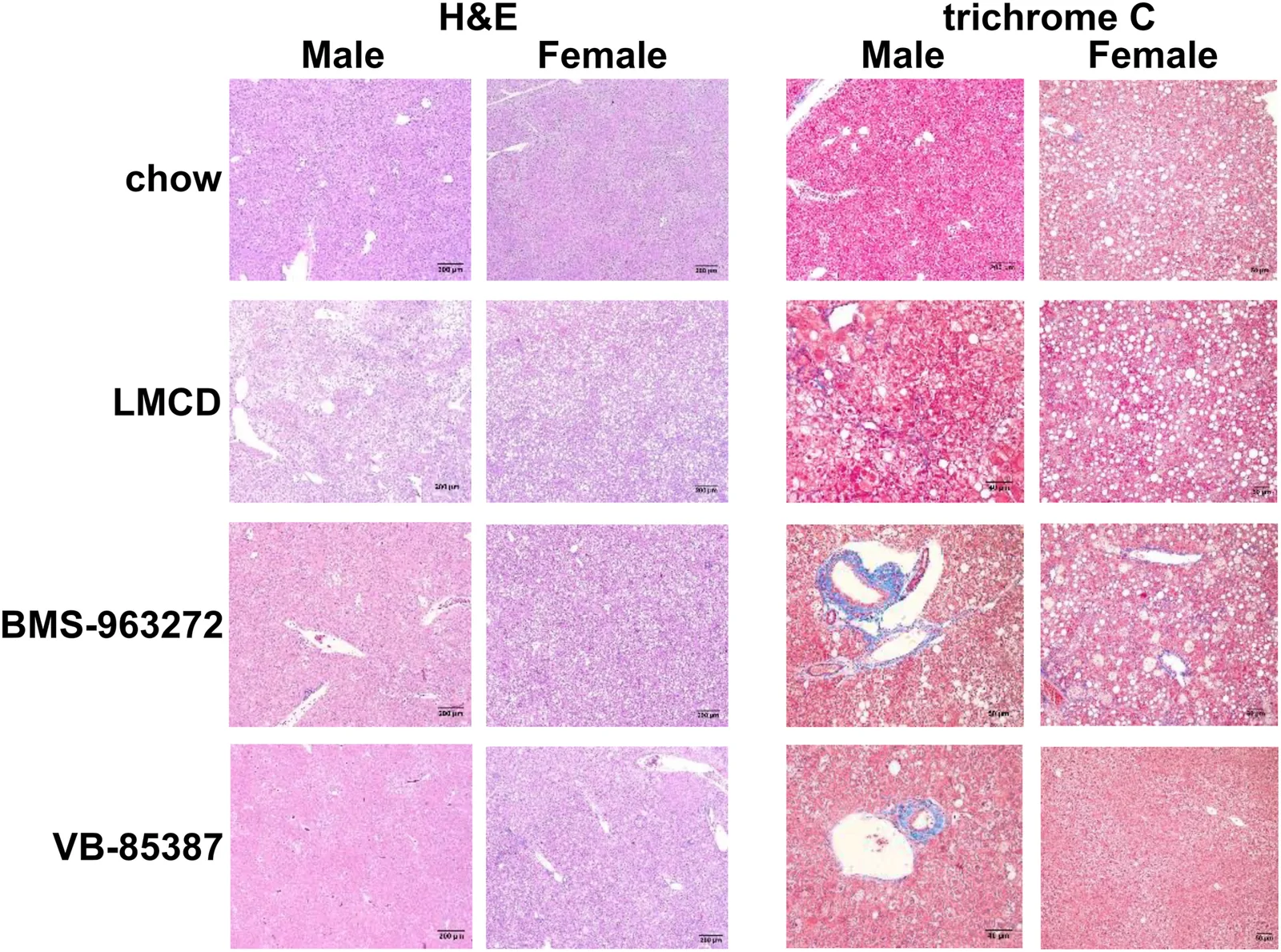
VB-85387 attenuates LMCD-induced hepatic steatosis and fibrosis in female mice. Liver samples from male and female mice underwent histological evaluation (n = 8). Slides were stained with H&E or Masson's trichrome C. LMCD, low methionine, choline-deficient diet; H&E, hematoxylin and eosin.

We previously showed that VB-85387 delays intestinal triglyceride transit using an oral lipid tolerance test in male mice (52). We determined whether female mice exhibit a similar triglyceride transit defect in the presence of varying concentrations of VB-85387 or 30 mg/kg comparator compound.

Female mice treated with VB-85387 showed a dose-dependent decrease in triglycerides transit compared to chow-fed mice (Fig. 3A, blue, purple, and orange circles), from 500 ug/dl at two 2 h in chow-fed mice to ∼300 at 30 mg/kg VB-85387. AUC analysis confirmed that all three doses of VB-85387 produced significant reduction in triglycerides (Fig. 3B). The comparator compound showed an equivalent reduction at 30 mg/kg.

**Fig. 3:**
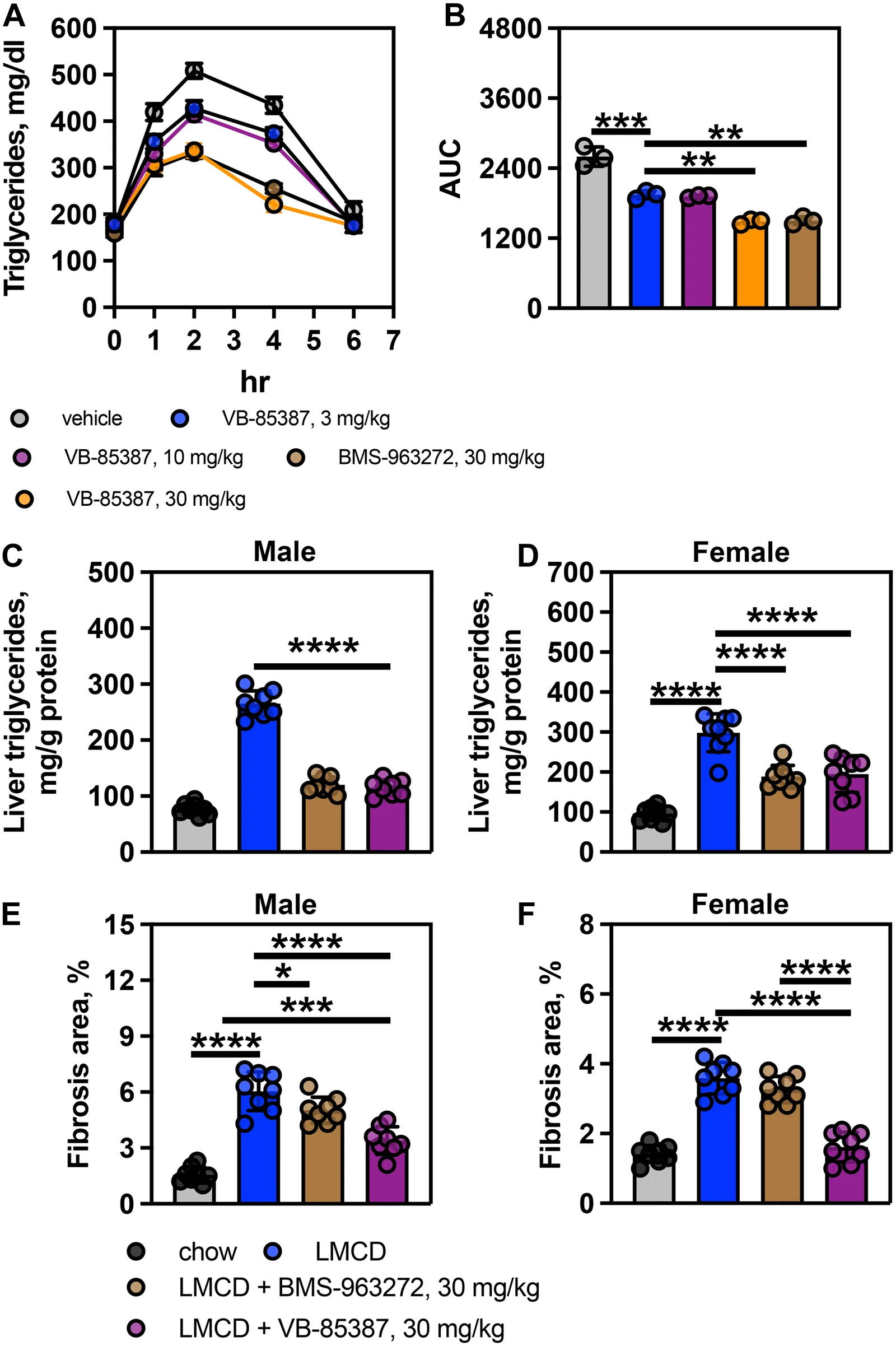
VB-85387 restores normal triglyceride levels in mice fed the LMCD diet. Male and female C57BL/6 mice were fed an LMCD diet as described in supplemental Fig. S1 (n = 8). A: A lipid tolerance test was performed on female mice. Mice were fasted and then administered olive oil by oral gavage. Blood triglyceride levels were measured at the indicated time points. B: AUC calculated from the results in panel A. C: liver triglyceride levels in male mice. D: liver triglyceride levels in female mice. E: Percentage fibrosis area in male mice. F: Percentage fibrosis area in female mice. Bioscience’s algorithm software was used to determine the percentage fibrosis area, with data calculated based on equal surface areas for all slides (57). Statistical analysis: two-way ANOVA with Tukey's post hoc test. Data are mean ± SD. ∗*P ≤ 0.01*; ∗∗*P ≤ 0.001*; ∗∗∗*P ≤ 0.0001*; ∗∗∗∗*P ≤ 0.00001*. LMCD, low methionine, choline-deficient; AUC, area under the curve.

Both male and female LMCD-fed mice accumulated hepatic triglycerides (Fig. 3C, D). Treatment with either VB-85387 or comparator reduced liver triglyceride levels in both cohorts (Fig. 3C, D). Computer-aided histological analysis showed that fibrosis area percentages increased in both male and female LMCD-fed mice (Fig. 3E, F). VB-85387 reduced fibrosis in both cohorts, whereas comparator was effective only in male mice. Thus, only VB-85387 could reduce fibrosis in both male and female mice.

To determine whether male and female mice fed the LMCD diet for 16 weeks exhibited altered intestinal and fecal distribution of free fatty acids and triglycerides, free fatty acid and triglyceride levels were measured in both male and female cohorts (Fig. 4A–H).

**Fig. 4:**
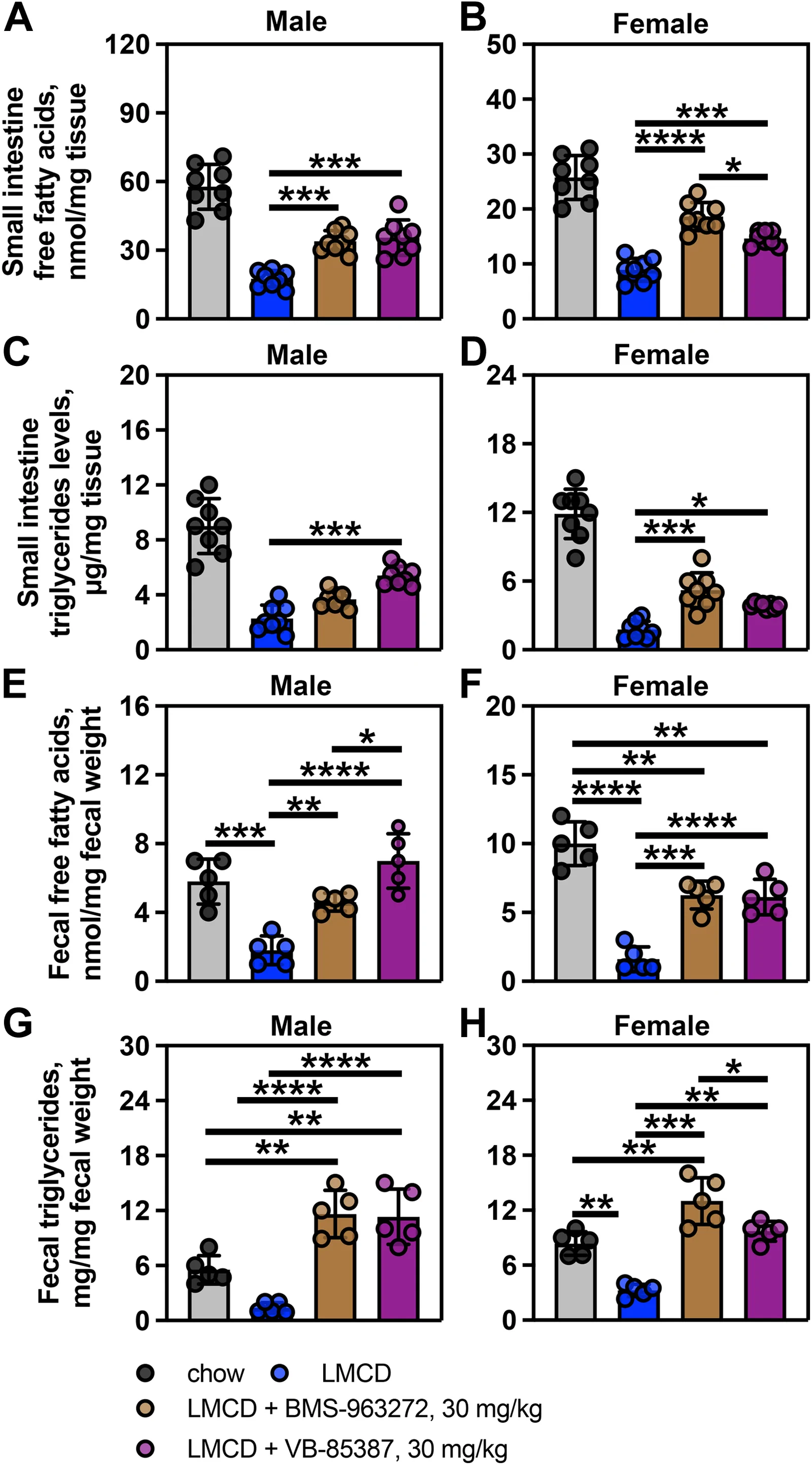
VB-85387 restores intestinal and fecal lipid homeostasis in LMCD-fed female mice. Small intestinal and fecal lipid levels were measured in LMCD-fed male and female mice (n = 8). A: Small intestinal free fatty acid levels in male mice. B: Small intestinal free fatty acid levels in female mice. C: Small intestinal triglyceride levels in male mice. D: Small intestinal triglyceride levels in female mice. E: Fecal free fatty acid levels in male mice. F: Fecal free fatty acid levels in female mice. G: Fecal triglyceride levels in male mice. H: Fecal triglyceride levels in female mice. Statistical analysis was performed using two-way ANOVA followed by Tukey's post hoc test. Data are presented as mean ± SD. ∗*P ≤ 0.01*; ∗∗*P ≤ 0.001*; ∗∗∗*P ≤ 0.0001; ∗*∗∗∗*P ≤ 0.00001*. LMCD, low methionine, choline-deficient.

Overall, male and female mice showed similar patterns of intestinal lipid distribution and comparable responses to treatment (Fig. 4A–H). The LMCD diet reduced intestinal free fatty acid and triglyceride levels in both sexes (Fig. 4A–D). Treatment significantly restored intestinal free fatty acid levels, although values did not reach those observed in chow-fed controls (Fig. 4A, B). The comparator compound was more effective at restoring free fatty acid levels in female mice (Fig. 4A vs. B). In contrast, intestinal triglyceride levels were restored by VB-85387, where the comparator was ineffective (Fig. 4C *versus* D).

Fecal free fatty acid levels were restored to near chow-fed levels by treatment with either compound (Fig. 4E, F). In male mice, VB-85387 and comparator increased fecal triglyceride levels above those observed in chow-fed controls (Fig. 4G, H), while VB-85387 was moderately less effective in female mice (Fig. 4G, H). Having determined that male and female mice generally benefited through VB-85387 treatment equally, we chose to use male mice for the longer term study.

### Mice on the LMCD diet typically exhibit lower body weight and decreased food consumption

We next used a chronic LMCD model in male mice to evaluate the effects of VB-85387 on MASLD/MASH progression after 24 weeks of feeding and 16 weeks of drug treatment (supplemental Fig. S2).

Body weights and food intake were measured at the specified times. Mice fed chow gained weight over time (29.26 ± 1.39 *vs*. 46.48 ± 0.92 g), while those on the LMCD diet (including LMCD + VB-85387 or comparator did not (24.3 ± 1.14 g and 26.6 ± 1.86 g, respectively) (Fig. S4A), consistent with previous observations (44, 45, 58). Daily food intake was similar across all LMCD cohorts and lower than chow-fed mice (2.47 ± 0.31 *vs*. 4.29 g) (supplemental Fig. S4B).

### VB-85387 treatment reduces histological evidence of hepatic steatosis and fibrosis in LMCD-fed mice

We evaluated VB-85387 efficacy by using histological staining to visualize the effect on hepatic steatosis in mice fed the LMCD for 24 weeks. Liver histology was assessed by a certified veterinary pathologist.

Mice maintained on a standard chow exhibited normal hepatocyte morphology with minimal evidence of microvesicular or macrovesicular steatosis (Fig. 5A, chow). Mice administered the LMCD developed pronounced hepatic steatosis, characterized by the accumulation of both microvesicular and macrovesicular steatotic lipid-laden cells (Fig. 5A, LMCD). Treatment of LMCD-fed mice with VB-85387 resulted in significant protection from hepatic steatosis (Fig. 5A). Protection was also observed in mice fed the comparator.

**Fig. 5:**
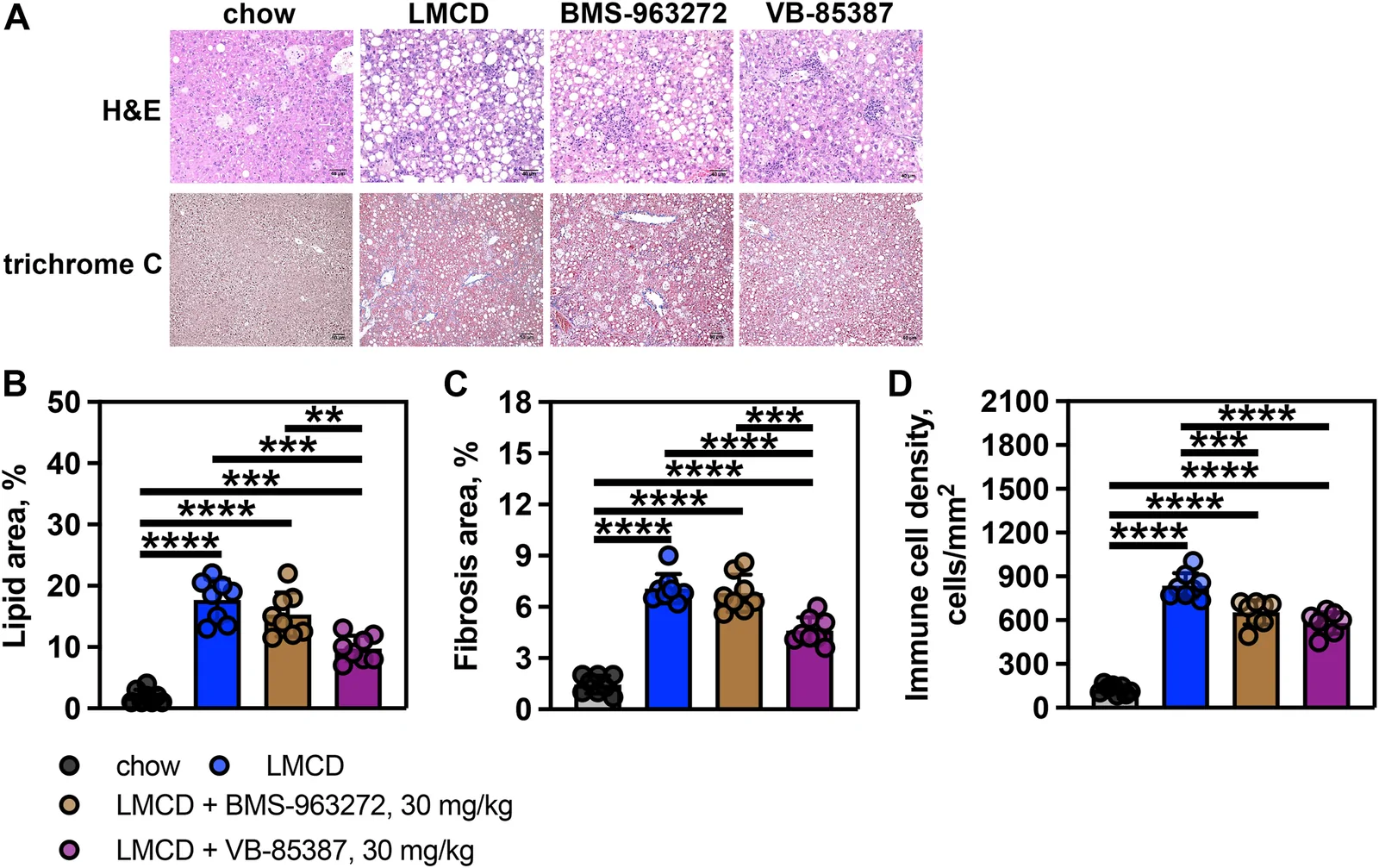
VB-85387 reduces MASLD and fibrosis in LMCD-fed mice. Liver samples from each cohort were subjected to histological evaluation (n = 8). Tissue sections were stained with H&E or Masson’s trichrome C. Quantitative analysis of stained sections was performed using Reveal Bioscience’s algorithm software (57), with data calculated based on equal surface areas for all slides. A: H&E and Masson’s trichrome C staining of liver tissue sections. B: Percentage lipid area. C: Percentage fibrosis area. D: Immune cell density. Statistical analysis was performed using two-way ANOVA followed by Tukey’s post hoc test. Data are presented as mean ± SD. ∗∗*P ≤ 0.001*; ∗∗∗*P ≤ 0.0001*; ∗∗∗∗*P ≤ 0.00001*. MASLD, metabolic dysfunction-associated steatotic liver disease; LMCD, low methionine, choline-deficient; H&E, hematoxylin and eosin.

Trichrome-C staining revealed mice given the LMCD exhibited signs of fibrosis. Mice treated with VB-85387 showed significant protection (Fig. 5A, LMCD *vs*. VB-85387).

To quantify the degree of fat and fibrosis accumulation computer-aided algorithmic software was used to determine the percentages of lipid and fibrosis areas (57). Mice maintained on chow exhibited minimal lipid accumulation. As expected this was markedly elevated in mice fed the LMCD over chow-fed mice (1.3 ± 0.35% *vs*. 17.5 ± 0.62%) (Fig. 5B, chow *vs*. LMCD). Administration of either VB-85387 or comparator led to a statistically significant reduction in the percentage of fat deposition; VB-85387-treated mice were almost twice as protected than mice fed the comparator compound (9.7 ± 0.68% *vs*. 15.7 ± 0.35%) (Fig. 5B, VB-85387 *vs*. BMS-963272).

As expected, administration of the LMCD induced significant fibrosis (1.5 + 0.83% vs. 7.5% *vs*. 1.5% (Fig. 5C, chow *vs*. LMCD). Treatment with VB-85387 resulted in a 50% reduction in LMCD-induced fibrosis (Fig. 5C, VB-85387 vs. LMCD). Interestingly, the comparator compound was not protective (Fig. 5C, BMS-963272 *vs*. LMCD).

Lipotoxicity triggers meta-inflammation, drawing immune cells to the liver (37). Dysfunctional adipose tissue also releases adipokines that attract macrophages, increasing immune cell infiltration (38). Chow-fed mice showed a low density of immune cell infiltration (Fig. 5D). Mice fed the LMCD had a 6.7-fold increase relative to chow-fed mice (Fig. 5D). Treatment with VB-85387 decreased immune cell density by 2.5-fold (Fig. 5D). The comparator compound was also able to decrease immune cell density.

### VB-85387 treatment reduces NASH activity scores in the LMCD model

Histological staging of steatosis, inflammation, and hepatocyte ballooning was performed to calculate NAS values for each cohort (Fig. 6A-D). VB-85387 treatment significantly reduced the number of mice exhibiting steatosis (Fig. 6A) and inflammation (Fig. 6B) compared with comparator-treated mice.

**Fig. 6:**
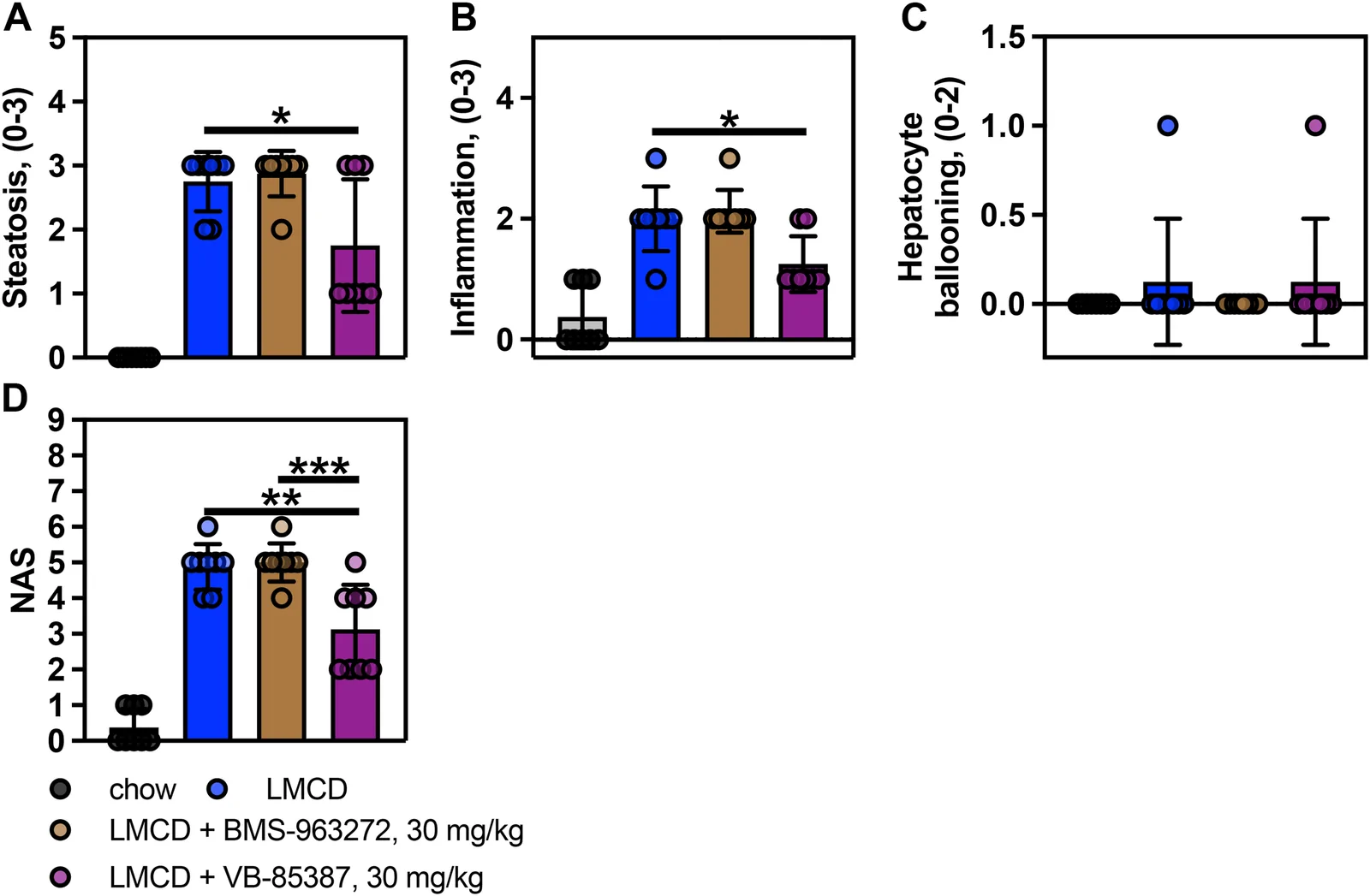
VB-85387 lowers NAS scores in LMCD-fed mice. NAS scoring was performed by a certified veterinary pathologist. A: Steatosis scores. B: Inflammation scores. C: Hepatocyte ballooning scores. D: NAS (n = 8). Statistical analysis was performed using two-way ANOVA followed by Tukey’s post hoc test. Data are presented as mean ± SD. ∗*P ≤ 0.01*; ∗∗*P ≤ 0.001*; ∗∗∗*P ≤ 0.0001*. NAS, NAFLD activity score; LMCD, low methionine, choline-deficient.

Chow-fed mice had a mean NAS of 1, whereas LMCD-fed mice had a mean NAS of 5, with 6 of 8 mice scoring at or above the MASH threshold (Fig. 6D). Comparator-treated mice showed a similar distribution of elevated NAS values. In contrast, only one VB-85387-treated mouse had a NAS of 5, while the remaining animals scored below the MASH threshold, indicating that VB-85387 reduced disease severity in the LMCD model.

### Fibrosis is reduced by VB-85387

Histological fibrosis staging was also performed. LMCD feeding significantly increased the number of mice showing evidence of fibrosis compared with chow-fed controls (supplemental Fig. S5A). This increase was further enhanced in LMCD-fed mice treated with the comparator compound. In contrast, the number of mice showing fibrosis after VB-85387 treatment was not significantly different from that observed in chow-fed mice, indicating that VB-85387 reduced fibrosis in the LMCD model (supplemental Fig. S5A).

### VB-85387 lowers liver triglycerides levels in mice fed the LMCD

One of the key pathological features of MASLD that is repeated in the LMCD is the elevation of liver triglycerides in the absence of therapeutics (59, 60). Normalizing liver triglycerides is a key therapeutic target in MASLD (61).

Since triglyceride accumulation is one of the key characteristics of the LMCD (62), we measured hepatic and serum triglycerides and cholesterol, as well as serum VLDL, LDL, and HDL levels.

Liver triglyceride levels were three times higher in mice fed the LMCD diet compared to those on standard chow (Table 2). Treatment with VB-85387 reduced triglyceride levels in LMCD-fed mice by 32%, while the comparator lowered them by 30%. Liver cholesterol levels remained consistent across all groups regardless of diet or treatment (Table 2).

**Table 2:** Serum and liver lipid levels in mice fed the LMCD

Lipid	Chow	LMCD	LMCD + VB-85387	LMCD + BMS-963272
Liver cholesterol mg/g protein	13.0 ± 2.8	12.2 ± 0.56	13.6 ± 1.7	15.4 ± 1.5
Liver triglycerides mg/g protein	108.1 ± 17.3	321.4 ± 22.6^a^	220 ± 15.8^c^	225.4 ± 20.4^c^
Serum cholesterol, mg/dL	128.6 ± 11.3	51.37 ± 11.7^a^	133.8 ± 10.8	140.6 ± 12.2
Serum triglycerides, mg/dL	141.6 ± 21.1	75.37 ± 14.2^a^	119.3 ± 14.3^b^	105.9 ± 10.7^b^
VLDL, mg/dL	3.75 + 0.76	0.94 + 0.23^a^	1.97 + 0.65^c^	2.81 + 0.57^c^
LDL, mg/dL	41.25 ± 10.8	35 ± 8.1	41.48 ± 5.7	30.12 ± 5.2
HDL, md/dL	97.5 + 13.6	15.2 + 3.1	95.7 + 15.9^a^	107 + 15.8^a^

n = 8.

Compared to mice fed the LMCD.

a *P* ≤ 0.0001.

b *P* ≤ 0.001.

c *P* ≤ 0.01.

Mice fed the LMCD diet showed a 1.9-fold reduction in serum triglyceride levels and a 2.5-fold reduction in serum cholesterol levels compared with chow-fed mice (Table 2). Treatment with VB-85387 and the comparator restored serum triglyceride levels to approximately 81% and 75%, respectively, of those observed in chow-fed mice. Moreover, serum cholesterol levels in LMCD-fed mice treated with VB-85387 returned to levels observed in chow-fed animals. Thus, VB-85387 was highly effective at reducing fatty liver under steatotic conditions, while the comparator compound was only partially effective.

### VB-85387 restores VLDL and HDL levels in mice fed an LMCD diet

Serum levels of VLDL, LDL, and HDL were also determined (Table 2). VLDL concentrations were reduced by four-fold in mice fed the LMCD diet compared to those fed chow. Treatment with VB-85387 restored VLDL levels to those observed in chow-fed controls. LDL concentrations remained unchanged in LMCD-fed mice relative to chow-fed animals; however, a significant reduction in LDL was noted in VB-85387-treated mice compared to those treated with comparator. HDL levels declined markedly by approximately 6.5-fold in LMCD-fed mice relative to chow-fed mice. Mice administered VB-85387 or comparator demonstrated restoration of HDL to normal levels. Thus, VB-85387 may reduce fatty liver, in part, by increasing VLDL secretion and promoting triglyceride export from the liver, thereby restoring normal triglyceride homeostasis.

### VB-85387 partially restores liver enzyme levels in LMCD-fed mice

Mice and humans with MASLD exhibit increased levels of alanine aminotransferase (ALT) and aspartate aminotransferase (AST) (63) (supplemental Table S1). These enzyme levels were measured.

LMCD-fed mice exhibited elevated ALT and AST levels compared with chow-fed mice, consistent with liver injury (Table S1). ALT levels decreased in LMCD-fed mice treated with either VB-85387 or the comparator; however, only VB-85387 treatment reduced AST levels in LMCD-fed mice.

### VB-85387 reduces hydroxyproline levels in LMCD-fed mice

Hydroxyproline, a breakdown product of collagen released during extracellular matrix remodeling by activated hepatic stellate cells, accumulates as fibrosis progresses and is an established biomarker of fibrotic burden (64). Mice fed the LMCD diet had significantly higher hydroxyproline levels than chow-fed mice (1,346 ± 62.82 *vs*. 195.25 ± 22.25 μg/g protein) (supplemental Table S2), consistent with increased hepatic collagen deposition. Treatment with VB-85387 reduced hydroxyproline levels (900.5 ± 54.14 μg/g protein), whereas the comparator did not produce a comparable effect (1,337 ± 61.60 μg/g tissue). These findings indicate that VB-85387 reduces hepatic fibrosis and liver injury in the LMCD model.

### VB-85387 reduces the protein levels of type I and III collagens in mice fed the LMCD

COL1A1 and COL3A1 encode the major type I and type III collagen proteins, respectively, which are key components of fibrotic extracellular matrix accumulation (65). Therefore, COL1A1 and COL3A1 protein levels were measured to further assess the effect of VB-85387 on collagen production and fibrotic progression. αSMA levels were also evaluated, as αSMA is induced when hepatic stellate cells transition to a myofibroblastic phenotype that supports collagen synthesis and fibrosis (66).

Western blot analysis showed that chow-fed mice exhibited low basal levels of COL1A1 and COL3A1 proteins (Fig. 7A). In contrast, LMCD feeding caused marked increases in COL1A1, COL3A1, and αSMA protein levels (Fig. 7A). Mice treated with comparator compound retained high levels, while levels markedly dropped in those treated with VB-85387.

**Fig. 7:**
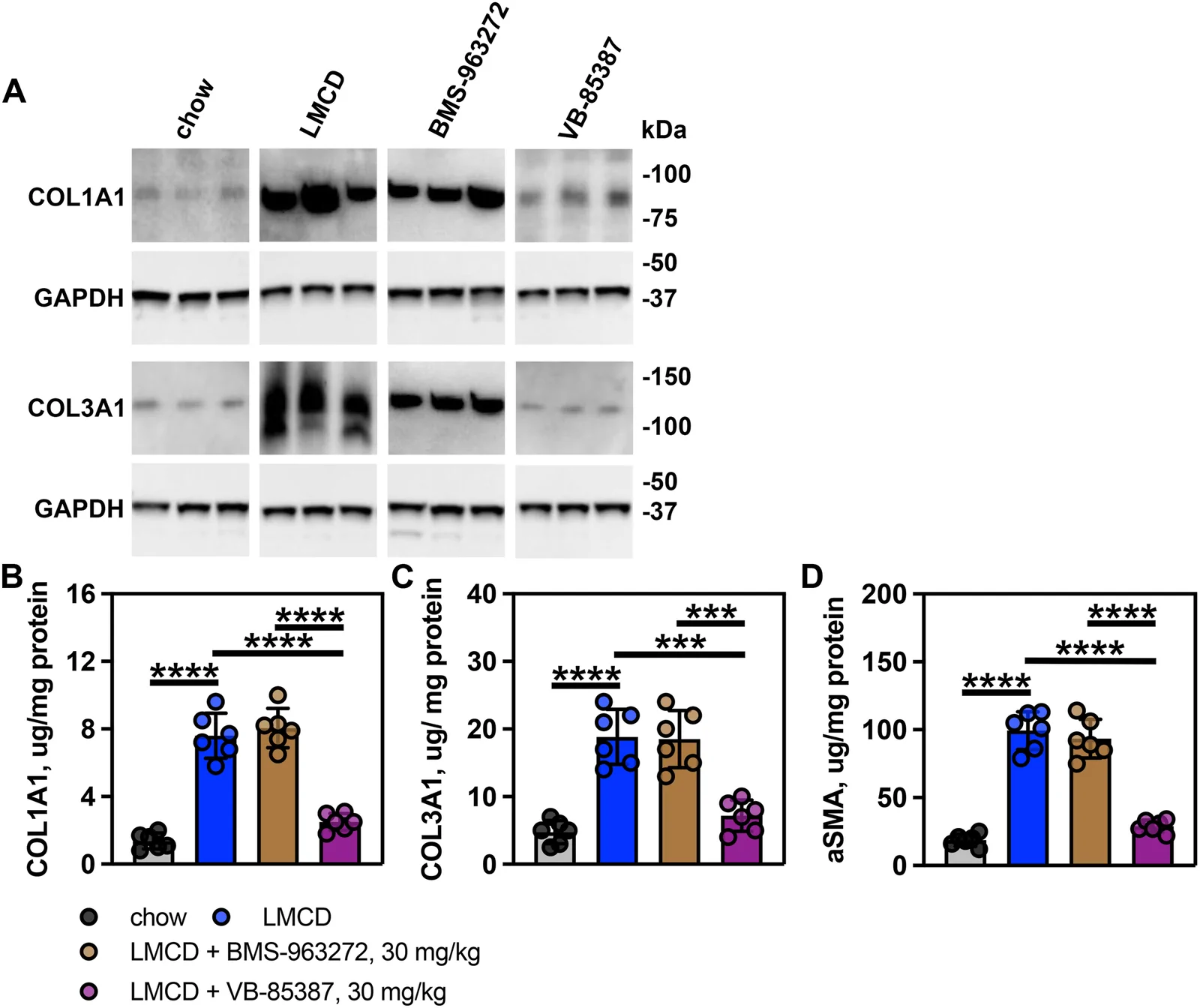
VB-85387 reduces type I and III collagen protein levels in mice fed the LMCD diet. Liver protein lysates were resolved on 4%–20% SDS-PAGE gels, transferred to nitrocellulose membranes, and probed with the appropriate antibodies. A: Western blot analysis of COL1A1 and COL3A1 protein levels in liver lysates, with GAPDH used as the loading control (n = 3).. The Western blot panels were generated from separate gels. For each gel, the protein of interest was blotted first; the membrane was then stripped and reprobed for GAPDH. B: COL1A1 levels determined by ELISA. C: COL3A1 protein levels determined by ELISA. D: αSMA levels determined by ELISA. Statistical analysis was performed using two-way ANOVA followed by Tukey’s post hoc test. Data are presented as mean ± SD. ∗∗∗*P ≤ 0.0001*; ∗∗∗∗*P ≤ 0.00001*. LMCD, low methionine, choline-deficient.

ELISA assays were used to quantify protein levels and confirmed the Western blot findings for COL1A1 and COL3A1 (Fig. 7B, C). Treatment with VB-85387 reduced both fibrosis-associated proteins, whereas the comparator was ineffective. ELISA analysis also showed elevated αSMA levels in LMCD-fed mice that was reduced by VB-85387 treatment alone (Fig. 7D). These findings indicate that VB-85387, but not the comparator, normalized LMCD-induced pathological protein elevations and reduced hepatic stellate cell activation.

### VB-85387 activates PPARα signaling

We previously showed that treatment of HFD-induced obese mice with VB-85387 induces Pparα signaling while reducing Srebp1-dependent transcription (52). PPARα activation promotes fatty acid β-oxidation and reduces hepatic fat accumulation, in part by increasing ketogenesis (67). In contrast, SREBP1 upregulates genes required for fatty acid synthesis and, ultimately, triglycerides synthesis (68). Therefore, we measured hepatic expression of Srebp1 and Pparα target genes to determine whether these pathways contribute to the ability of VB-85387 to limit hepatic triglyceride accumulation.

Mice fed the LMCD diet showed marked induction of the Srebp1-dependent genes *Srebf1*, *Acc1*, and *Fasn* (Fig. 8A). Treatment with VB-85387 restored expression of these genes to levels observed in chow-fed mice, and comparator compound produced a similar effect. In contrast, expression of the Pparα target genes *Ppara*, *Cpt1a*, and *Acot3* was markedly repressed in LMCD-fed mice compared with chow-fed controls (Fig. 8B). VB-85387 treatment induced expression of these genes above chow-fed levels, while the comparator also increased their expression and produced a greater induction of *Ppar*.

**Fig. 8:**
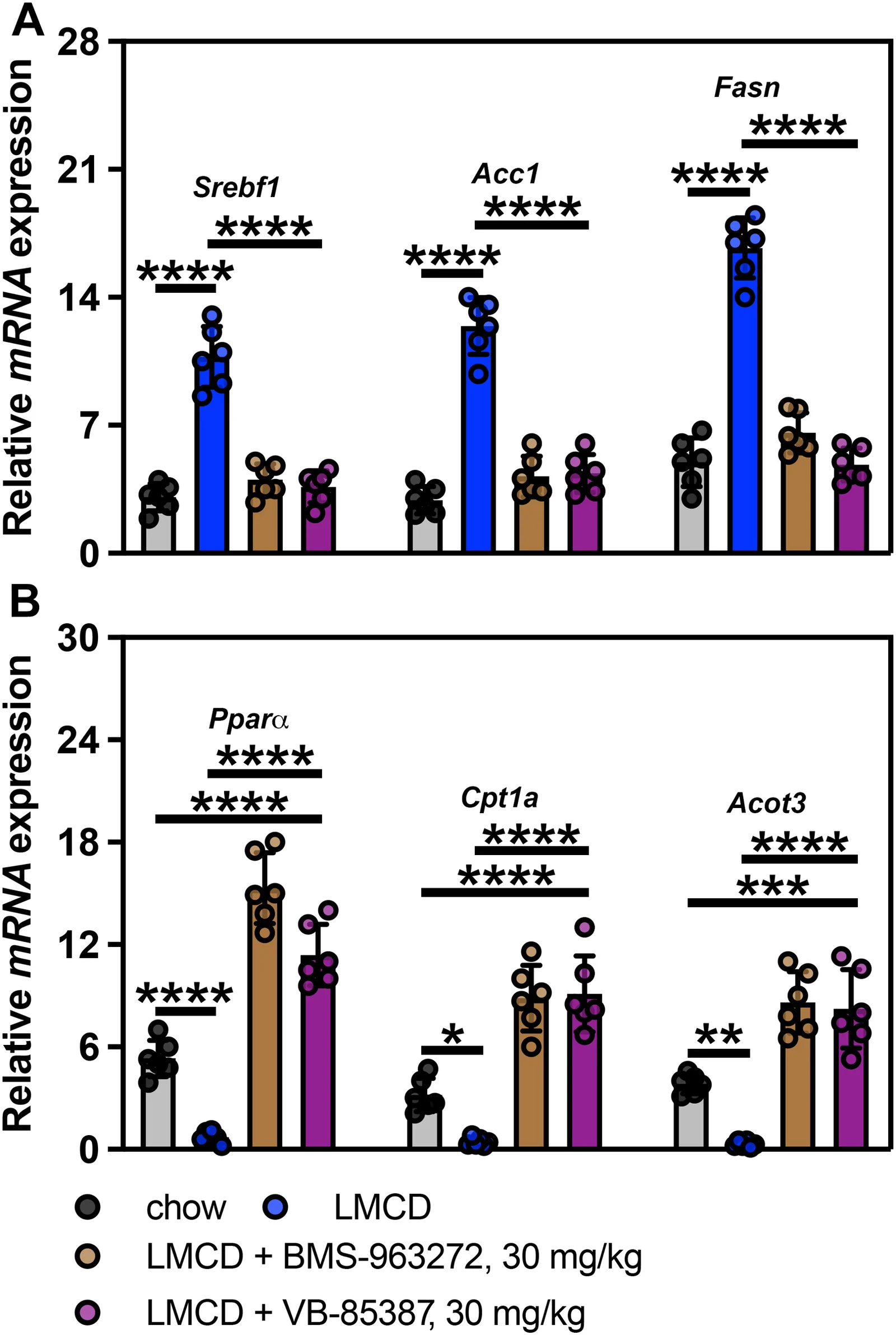
VB-85387 increases Pparα signaling and inhibits Srebp signaling. Total RNA was collected from liver tissues and used for determining gene expression levels by qRT-PCR (n = 8). A: Srebp1-dependent gene expression levels of *Srebf1*, *Acc1*, and *Fasn*. B: Pparα-dependent gene expression levels of *Pparα*, *Cpt1a*, and *Acot3*. Statistical analysis: two-way ANOVA with Tukey's post hoc test. Data are mean ± SD. ∗∗∗*P ≤ 0.0001*; ∗∗∗*P ≤ 0.0001*.

Together, these findings suggest that VB-85387 reduces MASLD, at least in part, by suppressing Srebp1-dependent fatty acid synthesis while enhancing Pparα-associated fatty acid β-oxidation.

### VB-85387 reduces adipocyte cell size and restores adipocyte cell number in LMCD-fed mice

Humans and mice with MASLD exhibit adipose tissue composed of fewer but larger adipocytes (60, 69). Epididymal adipose tissue was sectioned for histological analysis, stained with H&E, and examined via microscopy. Visual examination revealed that mice on the LMCD diet possessed larger adipocytes compared to those fed chow (Fig. 9A). Treatment with VB-85387 completely normalized adipocyte size as did the comparator.

**Fig. 9:**
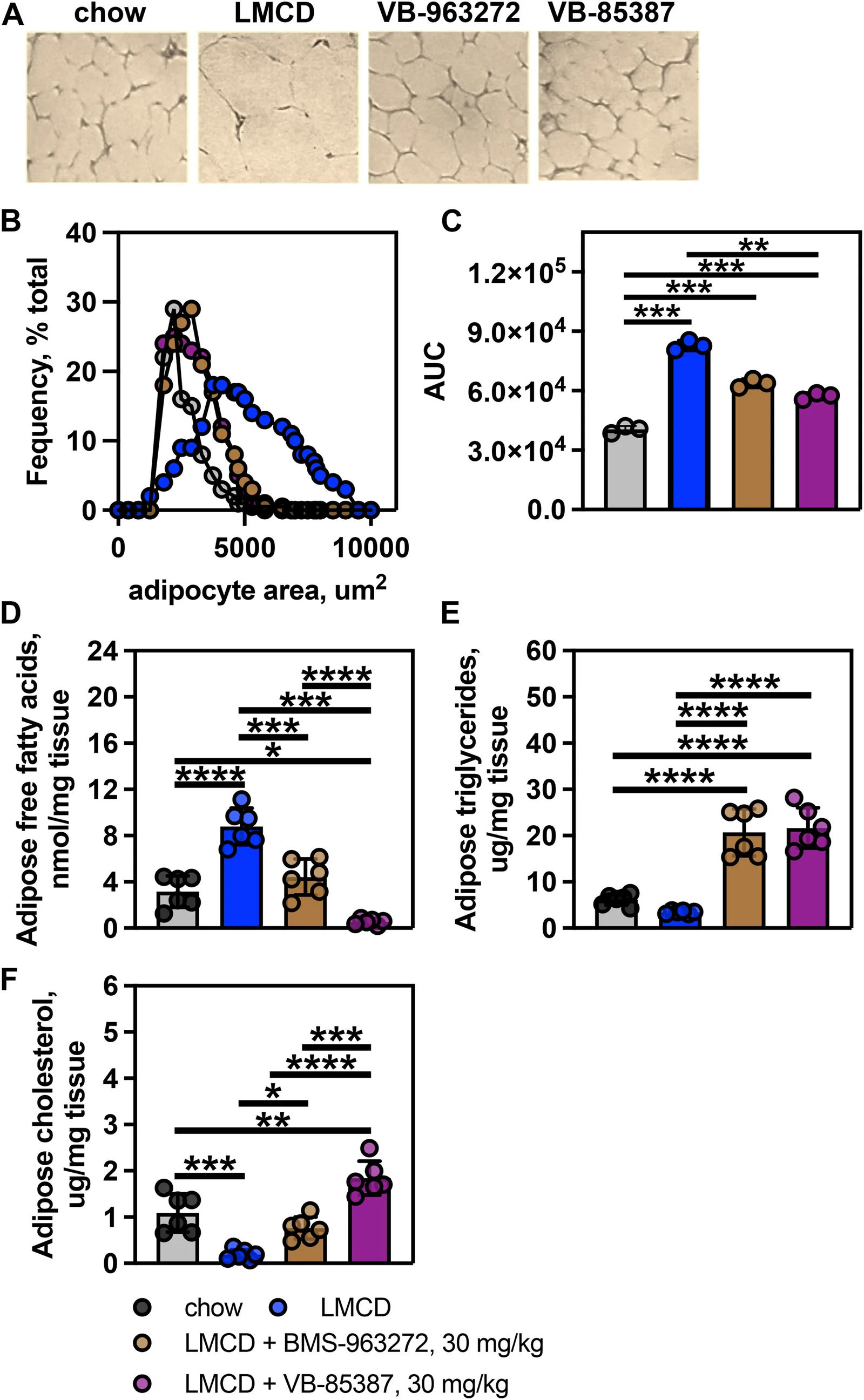
VB-85387 normalizes adipocyte size and lipid storage. Epididymal tissue was sectioned and stained with H&E. The micrographs shown are representative images from adipose sections of n = 8 mice. A: Adipose morphology. B: Image J software was used to measure the width of individual adipocytes. The measurements were compiled and used to plot variations in adipocyte cell area caused by drug and diet treatments. C: AUC values. D: Adipose tissue free fatty acids. E: Adipose tissue triglycerides. F: Adipose tissue cholesterol. Statistical analysis: two-way ANOVA with Tukey's post hoc test. Data are mean ± SD. ∗*P ≤ 0.01*; ∗∗*P ≤ 0.001*; ∗∗∗*P ≤ 0.0001*; ∗∗∗*P ≤ 0.0001*. H&E, hematoxylin and eosin; AUC, area under the curve.

ImageJ software was used to quantify individual adipocyte areas (51). Chow-fed mice had relatively smaller adipocytes than LMCD-fed mice, which exhibited a shift toward larger adipocytes (Fig. 9B, black circles vs. blue circles). LMCD-fed mice had markedly enlarged adipocytes whose size extended past 7,500 μM^2^. In contrast, adipocyte size distribution in VB-85387 mice much more closely resembled the normal adipocytes in chow-fed controls, as did the comparator (Fig. 9B, C, black circles vs. orange and purple circles).

To determine whether altered adipose lipid storage contributed to the increased adipocyte size observed in LMCD-fed mice, free fatty acids, triglycerides, and cholesterol levels were measured in epididymal adipose tissue. LMCD-fed mice exhibited a 2.2-fold increase in free fatty acid concentrations compared with chow-fed controls (Fig. 9D). VB-85387 lowered free fatty acid levels below those observed in chow-fed mice, while levels were restored to baseline in mice fed the comparator. Adipose triglyceride concentrations were similar in chow- and LMCD-fed mice; Treatment with VB-85387 or comparator increased triglyceride levels by 3.5-fold and 3.3-fold, respectively (Fig. 9E). Total cholesterol levels were reduced by 83% in LMCD-fed mice relative to chow-fed controls (Fig. 9F). VB-85387 increased cholesterol 1.7-fold compared with LMCD alone, while the comparator compound was markedly less effective.

Thus, VB-85387 restored adipocyte lipid homeostasis in LMCD-fed mice, which exhibited elevated free fatty acid levels and reduced triglyceride storage. These findings suggest that LMCD feeding promotes adipocyte lipolysis, potentially increasing free fatty acid delivery to the liver, and so contributing to hepatic triglyceride accumulation. VB-86387 was able to restore normal adipocyte lipid homeostasis.

### VB-85387 restores intestinal and fecal triglyceride and free fatty acid levels

It has been shown that mice lacking Mogat2 exhibit reduced intestinal triglyceride excursion while maintaining normal steady-state triglyceride levels, suggesting that Mogat deficiency may alter intestinal lipid transit and distribution (30). To determine whether VB-85387 produces sustained effects on intestinal and fecal lipid handling, free fatty acids, triglycerides, and cholesterol levels were measured.

Mice fed the LMCD showed reduced intestinal levels of less than 50% for all three lipid species (Fig. 10A–C). Treatment with VB-85387 restored free fatty acids levels to those observed in chow-fed mice (Fig. 10A). VB-85387 also restored intestinal triglycerides levels (Fig. 10B), and partially restored cholesterol levels, producing a 2.7-fold increase compared with levels in LMCD-fed mice (Fig. 10C).

**Fig. 10:**
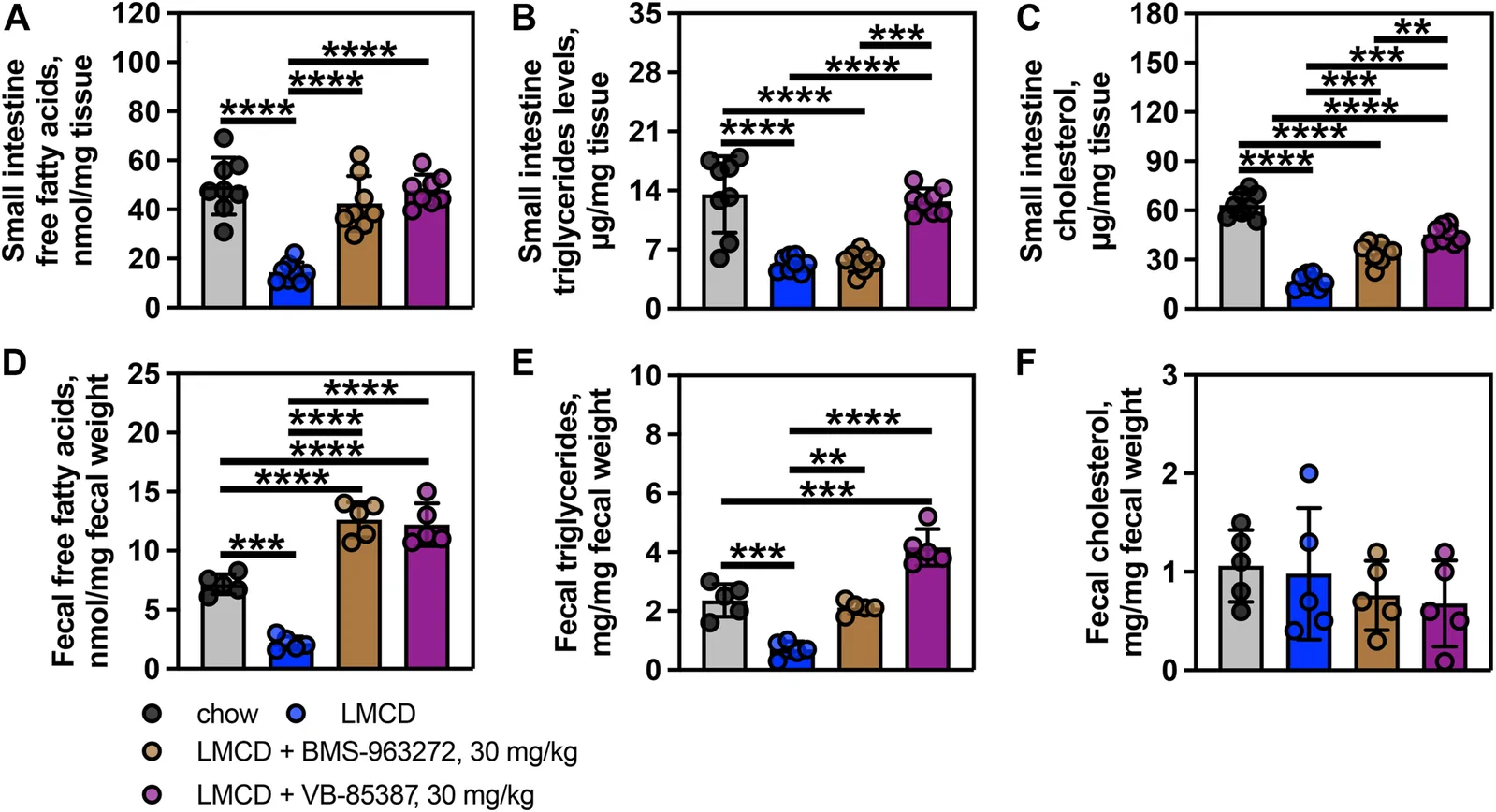
VB-85387 normalizes intestinal and fecal lipid profiles in LMCD-fed mice. Small intestinal and fecal lipid levels were measured in LMCD-fed mice (n = 8). A: Small intestinal free fatty acid levels. B: Small intestinal triglyceride levels. C: Small intestinal cholesterol levels. D: Fecal free fatty acid levels. E: Fecal triglyceride levels. F: Fecal cholesterol levels. Statistical analysis: two-way ANOVA with Tukey's post hoc test. Data are mean ± SD. ∗∗*P ≤ 0.001*; ∗∗∗*P ≤ 0.0001*; ∗∗∗∗*P ≤ 0.00001*. LMCD, low methionine, choline-deficient.

LMCD-fed mice exhibited reduced fecal free fatty acid and triglyceride levels, whereas fecal cholesterol levels remained unchanged (Fig. 10D–F). Treatment with VB-85387 overcame this effect of the LMCD, inducing a slight increase over that observed in chow-fed mice (2.1-fold) (Fig. 10D), and VB-85387 increased fecal triglycerides 2-fold above chow-fed levels (Fig. 10E).

Together, these findings indicate that VB-85387 alters lipid distribution by increasing fecal free fatty acid and triglyceride levels. The comparator compound mimicked the effects seen in VB-85387-treated mice, although it failed to restore small intestine triglycerides and only partially restored fecal triglycerides.

This pattern suggests that intestinal triglycerides are redirected toward fecal excretion, while free fatty acids may be generated within the intestinal lumen through pancreatic lipase-mediated triglyceride hydrolysis.

### STAM mice fed a HFD show reduced food intake

We next generated STAM mice by administering streptozotocin and feeding them a HFD, then evaluated the ability of VB-85387 to reduce diet-induced steatosis and fibrosis.

All groups treated with streptozotocin gained weight at rates comparable to chow-fed mice throughout the study, showing a 38% increase in body weight compared to baseline weights (supplemental Fig. S7A). This has been seen previously (19). Despite this, their food intake was significantly lower than that of chow-fed mice (supplemental Fig. S7B). Chow-fed mice consumed approximately 5.3 g/day, whereas STAM mice on a HFD or treated with VB-85387 or comparator ate about 1.6 g/day.

### VB-85387-treated mice show reduced histological signs of MASLD and fibrosis in HFD-fed STAM mice

We next used STAM mice to further assess the effects of VB-85387 on MASLD/MASH. The feeding and treatment regimen is detailed in supplemental Fig. S3.

H&E staining showed that STAM mice on a HFD developed hepatic steatosis with a higher percentage lipid area (Fig. 11A, B), which corresponded with higher liver triglyceride levels compared to chow-fed controls (Table 2). When HFD-fed STAM mice received VB-85387, histological evidence of steatosis was eliminated (Fig. 11A), and triglycerides levels fell to half those observed in chow-fed mice (Table 2). The comparator compound mimicked the effect of VB-86387 to a lesser extent.

**Fig. 11:**
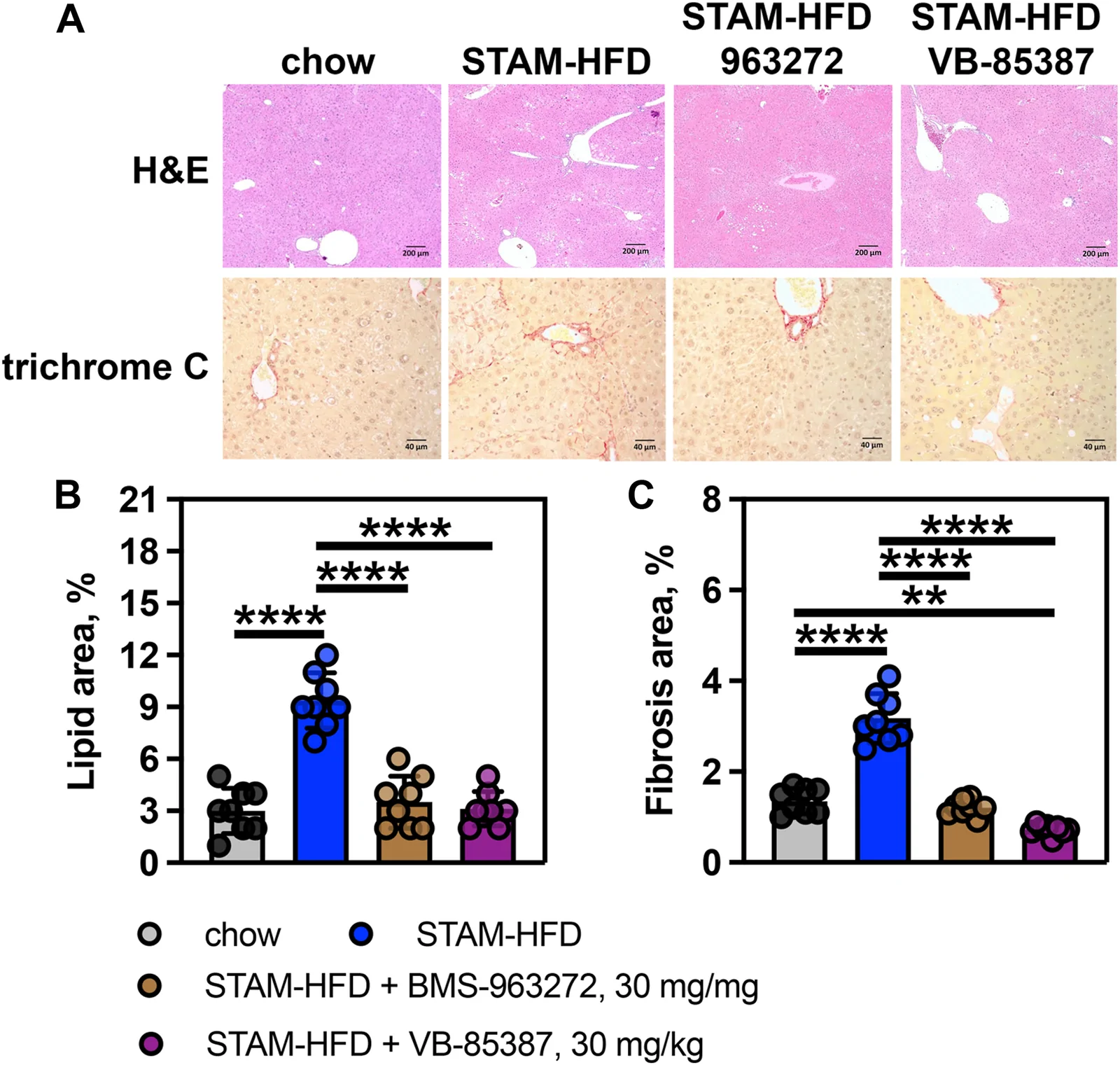
VB-85387 reduces MASLD and fibrosis in HFD-fed STAM mice. Male STAM mice were generated as described in supplemental Fig. S2. Mice were fed an HFD for 16 weeks and treated with VB-85387 (30 mg/kg) or cpmparator (30 mg/kg) (n = 8). Quantitative histological analysis of stained liver sections was performed using Reveal Bioscience’s algorithm software. A: H&E and trichrome C staining of liver tissue sections. B: Percentage lipid area. C: Percentage fibrosis area. Statistical analysis was performed using two-way ANOVA followed by Tukey’s post hoc test, with comparisons made to chow-fed mice. Data are presented as mean ± SD. ∗∗*P ≤ 0.001*; ∗∗∗∗*P* ≤ *0.00001*. MASLD, metabolic dysfunction-associated steatotic liver disease; HFD, high-fat diet; STAM, stelic animal model; H&E, hematoxylin and eosin.

STAM mice on a HFD showed a higher percentage fibrotic area than those on standard chow (Fig. 11C). VB-85387 reduced the percentage level of fibrosis to below that seen in chow-fed mice (Fig. 11C). VB-85387 lowered hydroxyproline levels by 30%, further indicating reduced fibrosis, while the comparator showed approximately half the therapeutic reduction (Fig. 11 remove (Fig. 11D), replace with supplemental Table S2.

Histological staging of steatosis, inflammation, and hepatocyte ballooning was performed in HFD-fed STAM mice (supplemental Fig. S6A–C). Although no mice in any cohort reached the NAS threshold for MASH development (NAS ≥5) (supplemental Fig. S6D), VB-85387 significantly reduced the number of mice with elevated NAS values compared with HFD-fed STAM mice. The comparator compound was less effective. VB-85387-treated mice also showed less signs of fibrosis compared to comparator compound (supplemental Fig. S5B).

### Liver enzymes levels are restored by VB-85387 treatment in STAM mice fed the HFD

Liver enzyme analysis showed elevated ALT and AST levels in STAM-HFD mice, consistent with liver dysfunction; however, treatment with VB-85387 restored both enzymes toward normal levels (supplemental Table S1).

### VB-85387 reduces NF-κB signaling and tumor necrosis factor alpha (TNFα) levels

The comparator compound has previously been shown to reduce the level of the key inflammatory cytokine, tumor necrosis factor alpha (TNFα) in STAM mice on a HFD (19). *Tnfα* expression is driven by NF-κB signaling via phosphorylation of its p65 subunit (70). A protein expression microarray containing key inflammation and apoptosis regulator proteins was used to examine signaling disruption in the STAM experimental diet and observe possible modifications by VB-85387 treatment.

Seventeen apoptosis biomarkers were examined (supplemental Fig. S8A). No effect was seen on levels of Atm, Casp3 (D175), Casp (D198), Chk1, Erk1/2, Hsp27, Jnk, p27, p38, and Smad, indicating their probable lack of involvement in MASLD/MASH development and pathology.

NFκB and IκBα were both elevated by the HFD, up to 3-fold (supplemental Fig. S8B). VB-85387 successfully ameliorated these diet-induced signaling mediators to the levels seen in chow fed mice. As, expected from previous reports, comparator behaved similarly (19). Interestingly, the induction diet also markedly elevated p53 and Tak11 levels, perhaps implicating them in the pathology of MASLD/MASH. VB-85387 completely reversed these elevations, restoring both p53 and Tak1 to chow-fed levels. Comparator failed to do this.

We further examined the expression and phosphorylation of NFκB and IκBα by Western blot. NFκB p65 levels were unchanged from chow diet levels by either the induction diet or by cotreatment with either VB-85387 or comparator. However, NFκB was markedly activated by the induction diet (Fig. 12A). Quantification of the relative activation between groups by scanning densitometry clearly shows the degree of activation of NF-kB by the induction diet and restoration to baseline activation by VB-85387. comparator approached, but did not meet, baseline levels (12B).

**Fig. 12:**
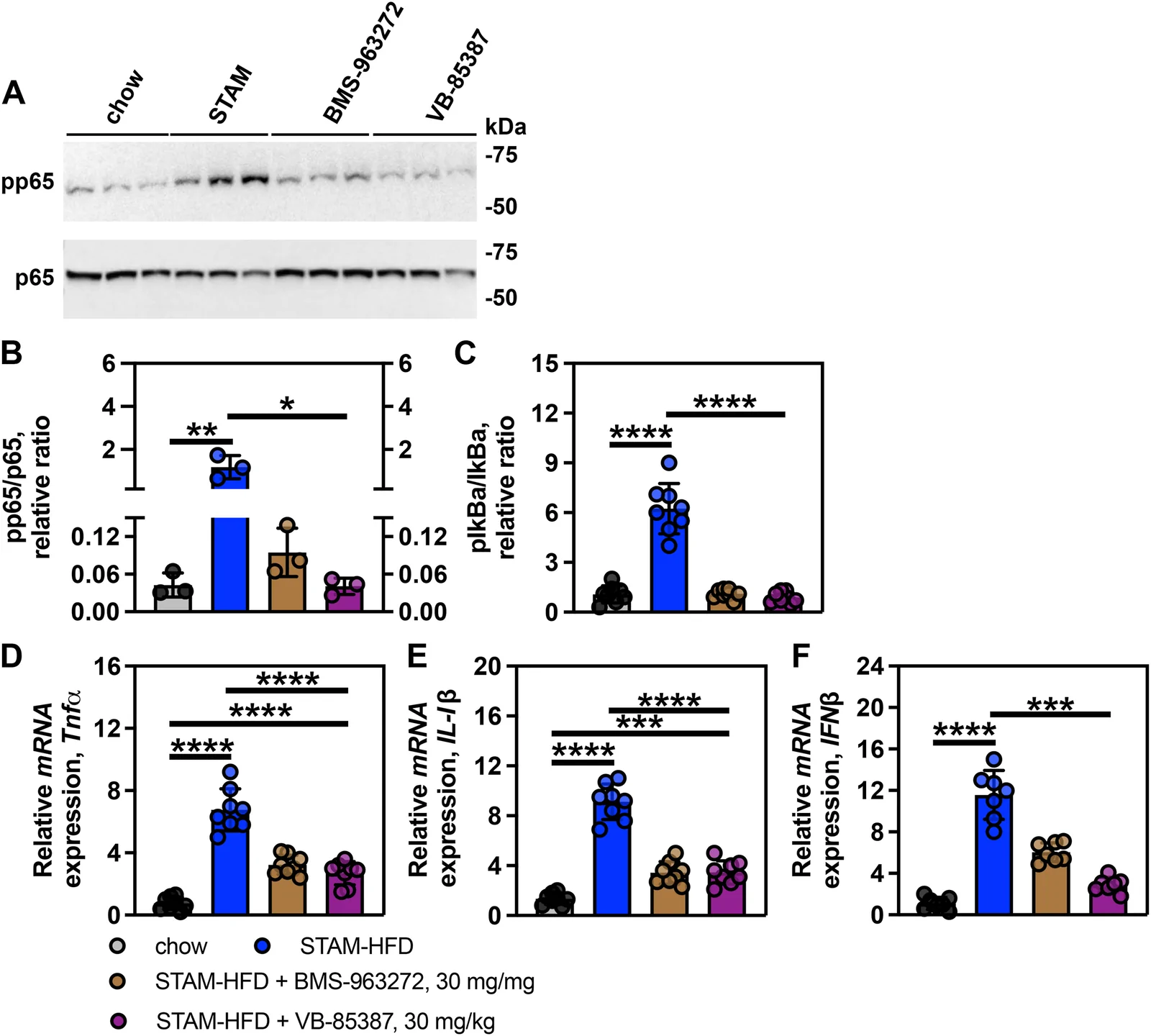
VB-85387 reduces NF-κB signaling in HFD-fed STAM mice. Liver tissue was used for all analyses. A: Phosphorylation levels of the p65 NF-κB subunit measured by western blotting. B: Relative pp65 levels calculated using ImageJ software (n = 3), with raw pp65 densitometry values normalized to total p65. C: Phosphorylated IκBα levels measured by ELISA (n = 8), with total IκBα levels used as baseline controls. D: Relative mRNA expression of *Tnfα*. E: Relative mRNA expression of *Il-1β*. F: Relative mRNA expression of *Ifnβ*. *Gapdh* mRNA expression was used as a control (n = 8). Statistical analysis was performed using two-way ANOVA followed by Tukey’s post hoc test. Data are presented as mean ± SD. ∗*P* ≤ 0*.01*; ∗∗*P ≤ 0.001*; ∗∗∗*P ≤ 0.0001*; ∗∗∗∗*P ≤ 0.00001*. NF-kB, nuclear factor kappa-light-chain-enhancer of activated B cells; HFD, high-fat diet; STAM, stelic animal model; TNFα, tumor necrosis factor alpha; Il-*1β*, interleukin-*1β*; Ifn*β*, interferon

Activation and therapeutic resolution of IκBα was quantified by ELISA. Once again, the induction diet showed a marked induction of phosphorylation of IκBα and complete therapeutic resolution by VB-85387. comparator behaved similarly.

To determine whether these changes in NFκB p65 activation were modifying the expression of proinflammatory cytokines, we used *q*RT-PCR to compare the expression of *Tnfα*, *Il-1β*, and *Ifnβ* under chow diet, induction diet, and VB-85387 treatment. As shown in Figure 12D–F, proinflammatory cytokine expression followed the activation states of NFκB exactly; expression levels were significantly elevated by the induction diet and resolved almost to baseline by VB-85387. Again, comparatorbehave similarly.

These findings indicate that VB-85387 reduces hepatic meta-inflammation that results from lipid accumulation in the liver, with a therapeutic profile that exceeds that of the comparator compound.

### VB-85387 successfully treats diet-induced Il-4 and Il-6 production with sequent downregulation of Jak1 activation

TNFα upregulates the expression of *Il-4* and *Il-6* (71, 72, 73). As might be expected elevated *Il-4* and *Il-6* levels have been observed in MASH patients (74). Furthermore, *Il-4* and *Il-6* combine to polarize primary human macrophages to a proinflammatory state (75), which may contribute to MASH pathology. We observed that *Il-4* and *Il-6* were amongst the cytokines elevated by induction of the HFD (supplemental Fig. S9). Therefore, we evaluated the effect of VB-85387 on their expression and on the activation of their initial signal transduction molecule, Jak1.

*Il-4* and *Il-6* levels were nearly undetectable in chow-fed STAM mice but increased almost tenfold in HFD-fed STAM animals (Fig. 13A, B). Treatment of HFD-fed STAM mice with VB-85387 reduced *Il-4* by 84%, almost back to baseline. comparator reduced it, but to a lesser extent (66%). (Fig. 13A). *Il-6* expression was reduced by over 50% by VB-85387, with the comparator compound behaving similarly (Fig. 13B).

**Fig. 13:**
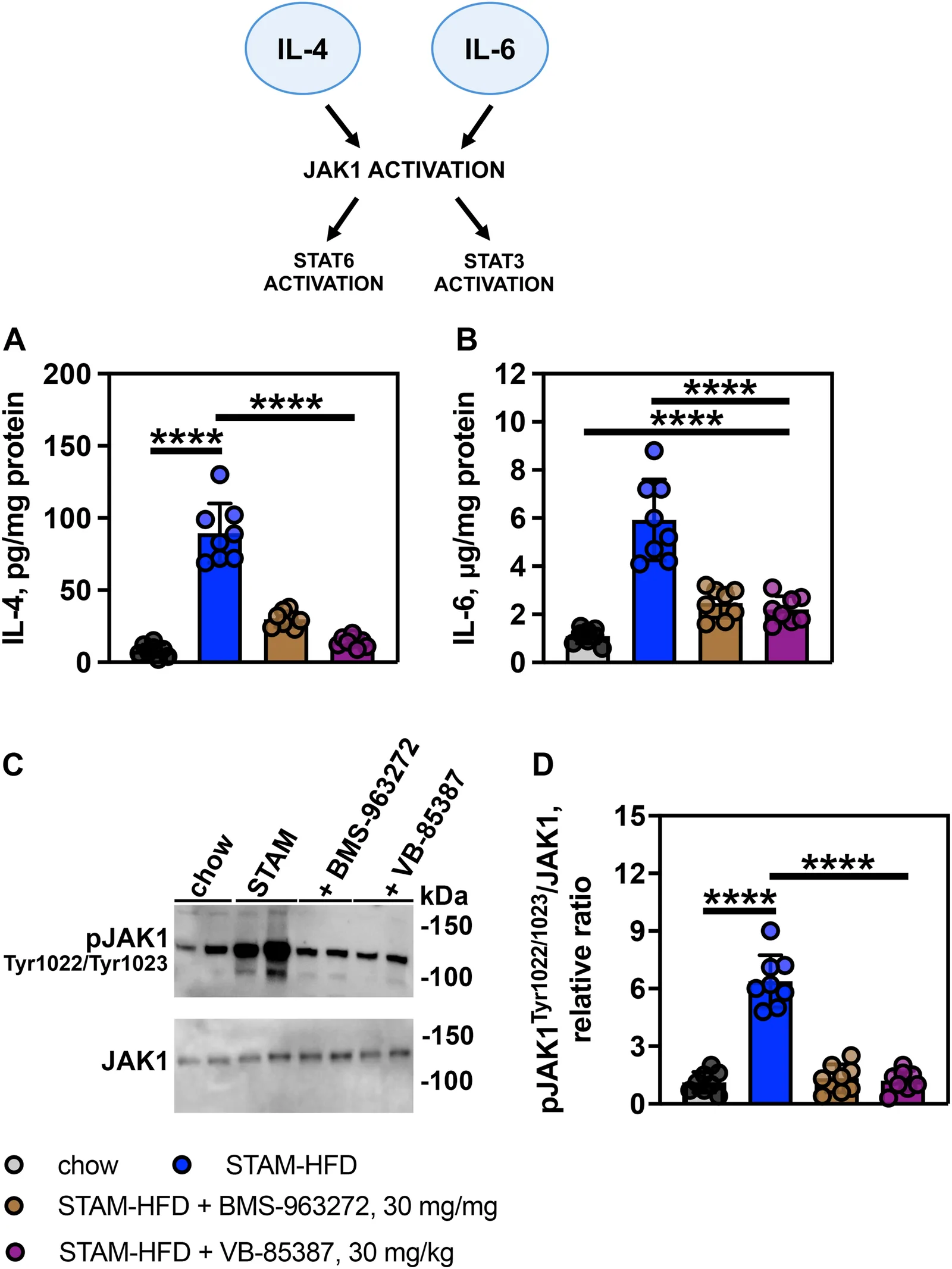
VB-85387 suppresses *IL-4*/*IL-6*-mediated JAK1 activation. Liver samples were analyzed by ELISA or western blotting, as indicated (n = 8). A schematic of *IL-4*/*IL-6*-mediated JAK1 activation is depicted. A: *IL-4* protein levels measured by ELISA. B: *IL-6* protein levels measured by ELISA. C: pJAK1^Tyr1022/Tyr1023^ and total JAK1 protein levels determined by Western blot analysis. D: Relative pJAK1^Tyr1022/Tyr1023^ levels normalized to total JAK1 using ELISA (n = 8). Statistical analysis was performed using two-way ANOVA followed by Tukey’s post hoc test. Data are presented as mean ± SD. ∗∗∗∗*P* ≤ *0.00001*. IL-4, interleukin-4; IL-6, interleukin-6; JAK, Janus kinase.

Jak1 can be activated by both *Il-4* and *Il-6*, so measure of Jak1 activation overall reflects the combined activity of both cytokines. Overall, Jak1 expression was constant in the 4 experimental groups (Fig. 13C). When we examined activated Jak1, it behaved as predicted from the patterns of *Il-4* and *Il-6* expression. Western blot analysis showed significant up regulation of phospho-Jak1 with the induction diet with a corresponding reduction under VB-85387 treatment and that of comparator. An ELISA assay determined that the induction diet upregulated Jak1 activation by over threefold, with the expected reduction to almost baseline levels was achieved by VB-85387 treatment and comparator (Fig. 13D).

## Discussion

MASLD/MASH progression is driven by interconnected metabolic, inflammatory, and fibrotic mechanisms (60, 76). Hepatic lipid accumulation initiates low-grade meta-inflammation (60), and disease progression is reinforced by cytokine and adipokine release (77, 78). TGF-β1 activates hepatic stellate cells (79), promoting collagen deposition and hepatic fibrosis (80). As inflammation intensifies, cytokine production recruits’ immune cells that release chemoattractants and direct cytotoxic T cells to sites of hepatic injury (81). Resident hepatic T cell populations are also activated during disease progression (82). Together, these processes promote hepatocyte death, liver scarring, and progression toward cirrhosis and hepatocellular carcinoma.

Mice fed an LMCD are known to develop MASLD and/or MASH, often accompanied by fibrosis (83). Treatment with either VB-85387 or BMS-963272 attenuated MASLD progression, lowered hepatic triglyceride levels, and reduced liver steatosis. VB-85387 also reduced fibrosis and collagen deposition, as indicated by lower collagen protein expression and hydroxyproline levels. Although all LMCD-fed groups developed MASH, the proportion of mice with NAS values above the MASH threshold was lower in the VB-85387-treated group than in the BMS-963272-treated group, whose NAS values exceeded this threshold and were comparable to those observed in untreated LMCD-fed mice.

Previous studies have shown that mice fed a choline-deficient amino acid-defined HFD (CDAA-HFD) exhibited reduced fibrosis after treatment with BMS-963272 at 3 mg/kg (19). At this dose, BMS-963272 significantly reduced liver fibrosis without altering hepatic triglyceride levels. In our study, we used a 10-fold higher BMS-963272 dose that showed reduced hepatic lipid area but no significant effect on fibrosis in LMCD-fed mice. The studies were performed under different experimental conditions, including differences in diet (LMCD vs. CDAA-HFD) and duration and length of drug treatment. It is possible that, with longer CDAA-HFD feeding, fibrosis might advance to the point where it can no longer be reduced by BMS-963272. However, we cannot make a definitive conclusion because differences in pharmacokinetic properties may substantially influence efficacy. The rapid absorption and lipophilicity of VB-85387 may allow it to persist longer in the intestine than BMS-963272. In contrast, BMS-963272 is a more hydrophilic molecule and may therefore have greater systemic exposure. In addition, the extent to which these compounds are metabolized by CYP enzymes is unknown and could also have a significant effect on efficacy. The prior study tested STAM mice maintained on a HFD, and their findings closely align with those reported here.

LMCD-fed mice had a higher proportion of enlarged adipocytes with lower triglyceride content and elevated free fatty acid levels. A greater number of smaller adipocytes with increased triglyceride storage were seen in mice treated with VB-85387. Such smaller, hyperplastic adipocytes are associated with improved glucose uptake and insulin sensitivity (84). Elevated free fatty acids levels in LMCD-fed mice may reflect increased lipolysis and adipocyte dysfunction (85). Circulating free fatty acids bind albumin and are transported into the liver through Cd36, Fatp2, and Fatp5, thereby increasing substrate availability for triglyceride synthesis and contributing to MASLD (86). Thus, one way VB-85387 may reduce hepatic triglyceride accumulation, and MASLD severity is by restoring adipocyte function and lipid homeostasis.

In most cases, VB-85387 treatment restored small intestinal and fecal free fatty acids and triglycerides levels in both male and female mice toward those observed in chow-fed controls in our 16 weeks study. In males treated with VB-85387 for 16 weeks, fecal triglyceride levels increased above those observed in males fed chow, although this effect was not observed in female mice. Nonetheless, however, our findings overall indicate that female mice responded similarly to male mice with respect to diet-induced changes in intestinal lipid transit. This result is consistent with studies showing that mice lacking *Mogat2* exhibit delayed triglyceride transit through the small intestine while maintaining normal steady-state triglyceride levels (87). Female mice also showed histological changes, elevated hepatic triglyceride levels, and early signs of fibrosis like those observed in male cohorts that was therapeutically resolved by treatment with VB-85387. Thus, both male and female mice showed comparable sensitivity to VB-85387 treatment.

Several findings indicate that VB-85387 dampens lipotoxic meta-inflammation. In HFD-fed STAM mice, NF-κB signaling was activated, as reflected by increased phosphorylation of p65 and IκBα. VB-85387 reduced NF-κB activation and lowered NF-κB-driven *Tnf*α expression, consistent with suppression of lipid-induced inflammatory signaling.

VB-85387 also reduced *Il-4* levels and Jak signaling, pathways that are known to contribute to MASLD/MASH progression. Together, these results suggest that VB-85387 may attenuate meta-inflammation by lowering hepatic triglyceride burden and suppressing downstream cytokine signaling.

*Il-4* activates JAK1 signaling, a pathway that can amplify *Il-4*-dependent inflammatory responses and promote downstream STAT-mediated activation. Jak1 has also been reported to phosphorylate insulin receptor substrate-1, thereby impairing insulin receptor-dependent signaling and contributing to insulin resistance. This mechanism enhances hepatic de novo lipogenesis, lipid accumulation, and meta-inflammation. In STAM mice, both VB-85387 and BMS-963272 reduced pJAK1 levels, indicating attenuation of *Il-4*-associated Jak1 signaling and its downstream inflammatory effects.

Il-6 expression is elevated in patients with MASLD and may contribute to disease progression by promoting inflammatory and fibrotic signaling. In HFD-fed STAM mice, elevated *Il-6* levels were associated with increased Crp, an *IL-6*-regulated inflammatory marker, consistent with ongoing systemic inflammation (supplemental Fig. S10). VB-85387 treatment reduced both *Il-6* and Crp levels, suggesting that it suppresses *Il-6*-associated inflammatory signaling in this model.

What mechanisms allow VB-85387 to attenuate MASLD and fibrosis? Monoacylglycerol (MAG) levels are closely linked to metabolic homeostasis. MAG is a substrate for monoacylglycerol lipase (MAGL), which hydrolyzes MAG to fatty acids and glycerol (88). Intestinal MAGL overexpression reduces MAG levels, increases food intake, decreases energy expenditure, and promotes obesity (89). In contrast, MAGL ablation reduces weight gain and delays lipid absorption, resulting in a lean phenotype (90). Loss of MAGL also increases energy expenditure (91), and MAGL-knockout mice preferentially consume normal chow rather than a HFD (90). MAG also functions as a ligand for the G protein-coupled receptor GPR119, which is highly expressed in intestinal enteroendocrine L cells (92). Activation of GPR119 stimulates GLP-1 and cholecystokinin release, thereby improving glucose tolerance (92). Storch *et al.* (93) have suggested that MAGs may be retained in signaling pools within intestinal enterocytes.

VB-85387 effectively resolved MASLD and fibrosis in both dietary models by lowering hepatic triglyceride levels. Although VB-85387 did not fully prevent MASH in all LMCD-fed mice, fewer treated mice had NAS values above the MASH threshold suggesting efficacy in lowering MASH severity. In HFD-fed STAM mice, both compounds resolved MASLD and reduced fibrosis severity. Overall, our results support VB-85387 as a strong candidate for further medicinal chemistry optimization and preclinical development as a potential therapy for MASLD and associated fibrosis.

## Conclusion

VB-85387 reduced MASLD and fibrosis in both LMCD-fed mice and streptozotocin-treated mice maintained on an HFD, with efficacy comparable to or greater than that of the preclinical MOGAT2 inhibitor BMS-963272. Although BMS-963272 reduced MASLD and lowered triglyceride levels in the shorter LMCD study in both male and female mice, it failed to reduce lipid area in the chronic LMCD study, whereas VB-85387 remained effective in both models. In the LMCD model, only VB-85387 reduced fibrosis and collagen deposition, suggesting that BMS-963272 does not adequately suppress hepatic stellate cell activation under these conditions.

Based on our results, we hypothesize that VB-85387 functions in vivo to inhibit intestinal MOGAT2, which reduces the levels of triglycerides which in turn reduces the level of hepatic steatosis. This hypothesis is supported by mouse studies showing that mice lacking Mogat2 (*Mogat2−/−)* are resistant to diet-induced hepatic steatosis (30). Induced expression of human MOGAT2 in the intestines of these mice restores the steatotic phenotype as well as others associated with diminished mMOGAT2 activity in *Mgat2*^*−/−*^ mice (31, 87). This leads us to propose that the inhibition of intestinal MOGAT2 will have profound therapeutic effects on globally elevated triglycerides and fatty acid levels thereby restoring triglycerides homeostasis leading to a reduction in hepatic steatosis and liver fibrosis resulting from increased hepatic stellate cell activation, collagen deposition, and proinflammatory signaling activation (meta-inflammation).

## Data availability

All data will be made available upon request to the corresponding author.

## Supplemental data

This article contains supplemental data.

## Conflict of interest

The authors declare the following financial interests/personal relationships which may be considered as potential competing interests. All authors are current or former employees of Genesis Biotechnology Group.

## Figures and Tables

**Fig. S1:**
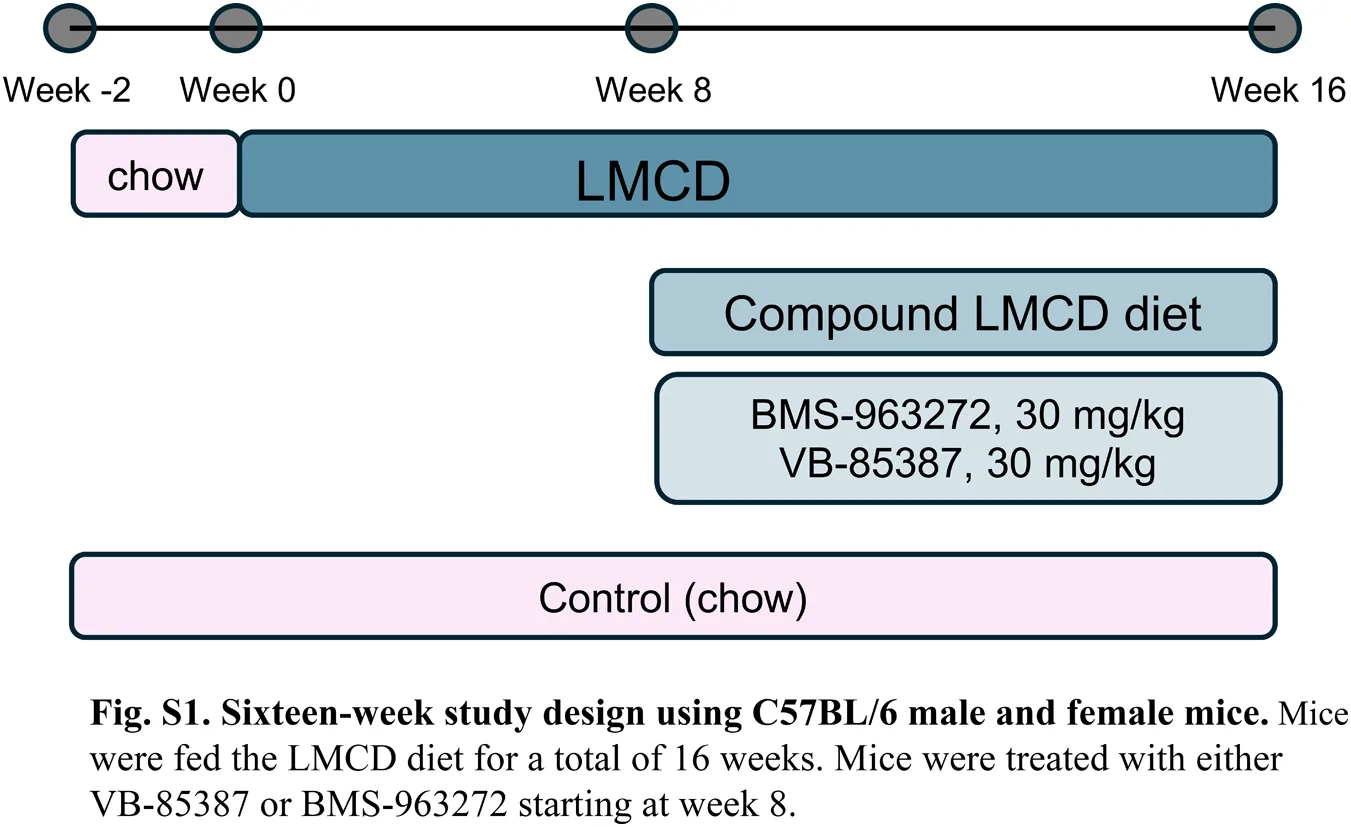


**Fig. S2:**
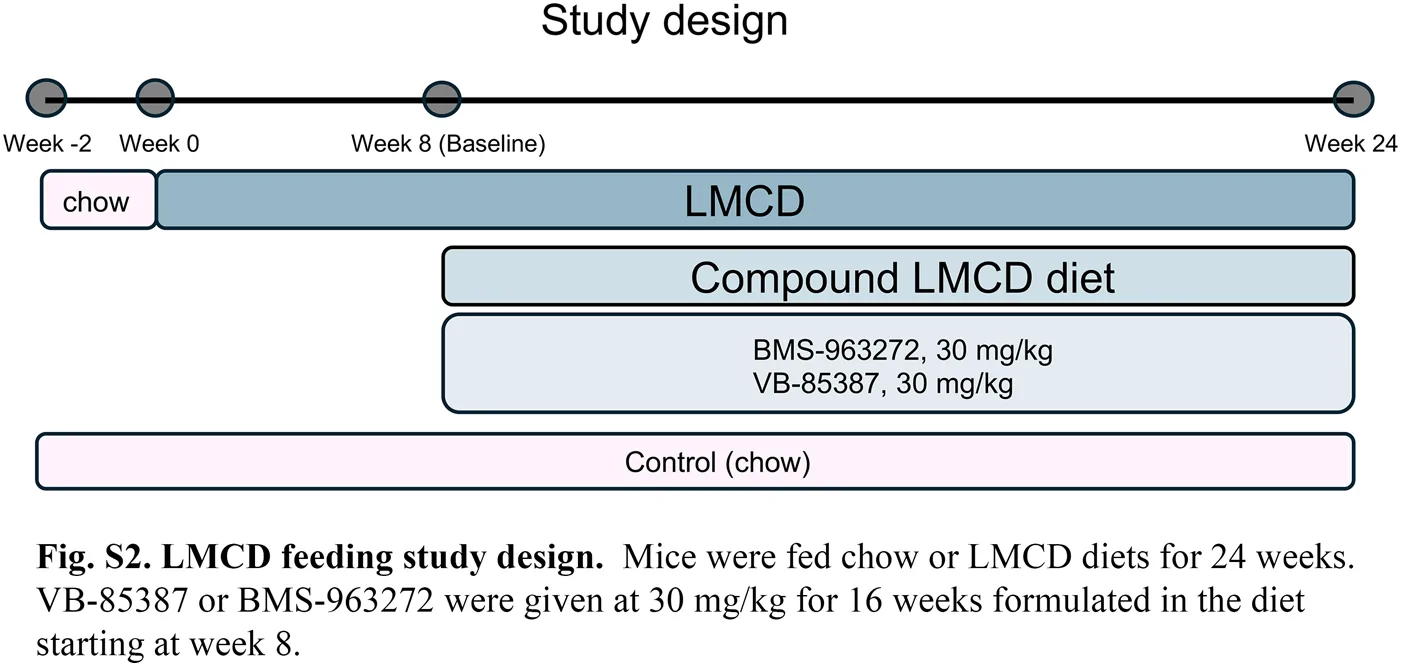


**Fig. S3:**
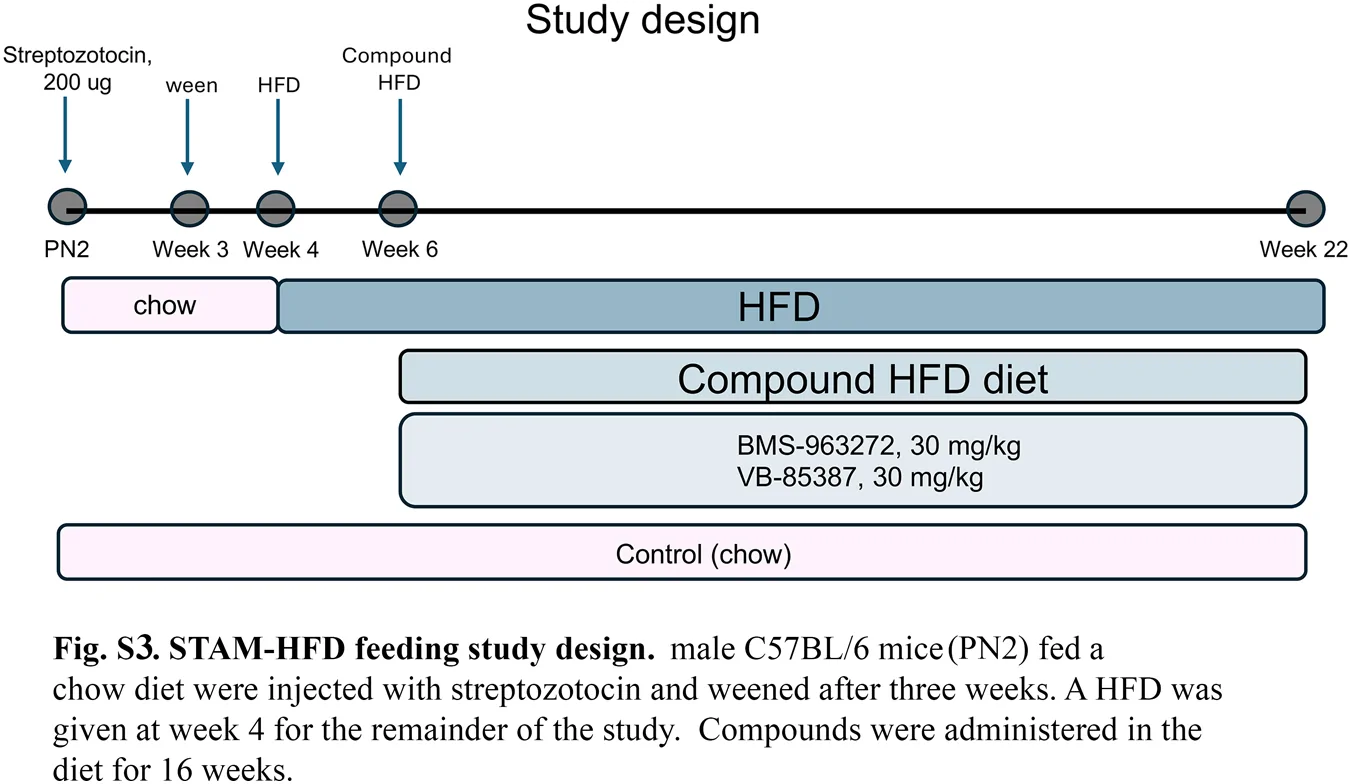


**Fig. S4:**
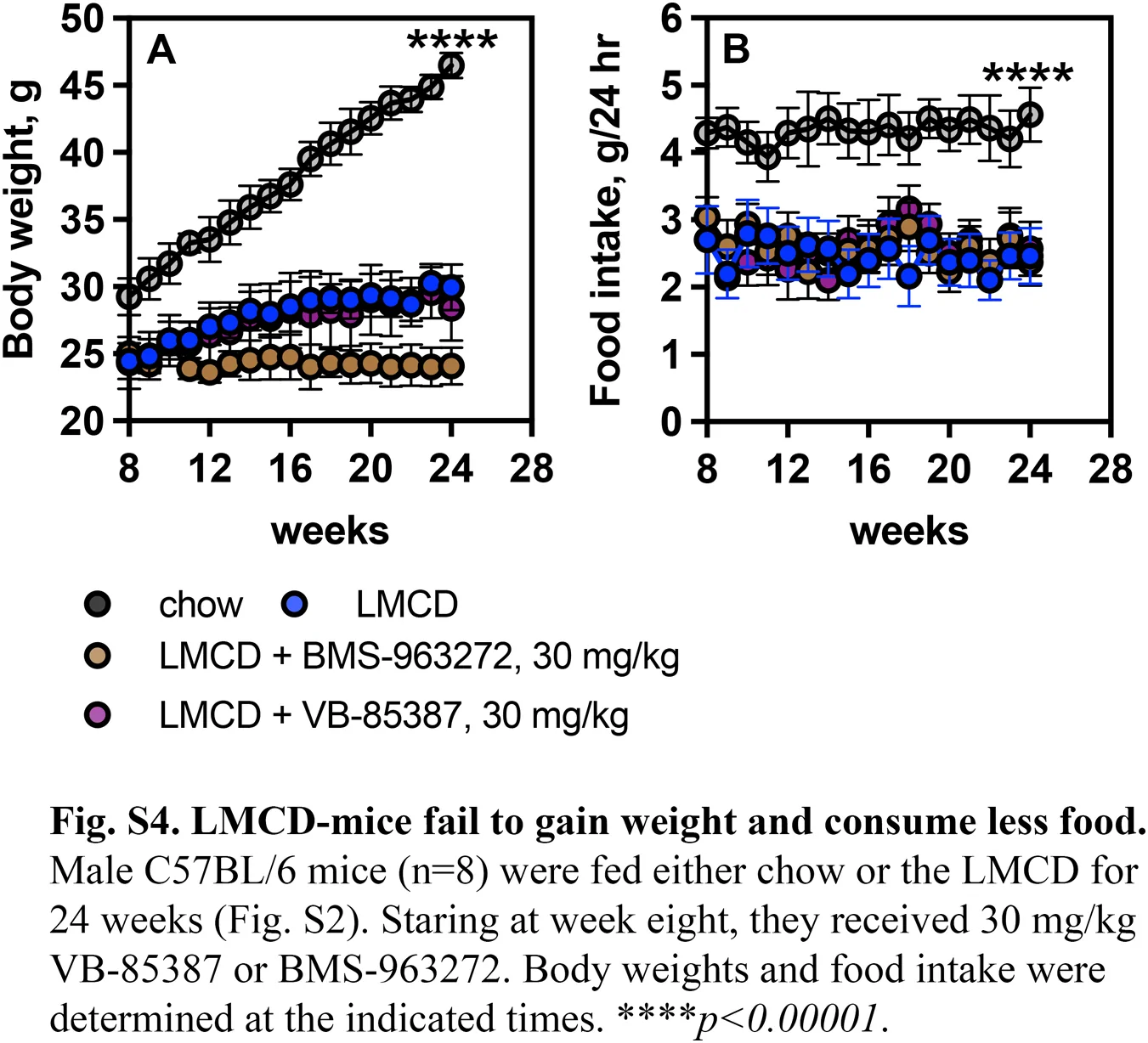


**Fig. S5:**
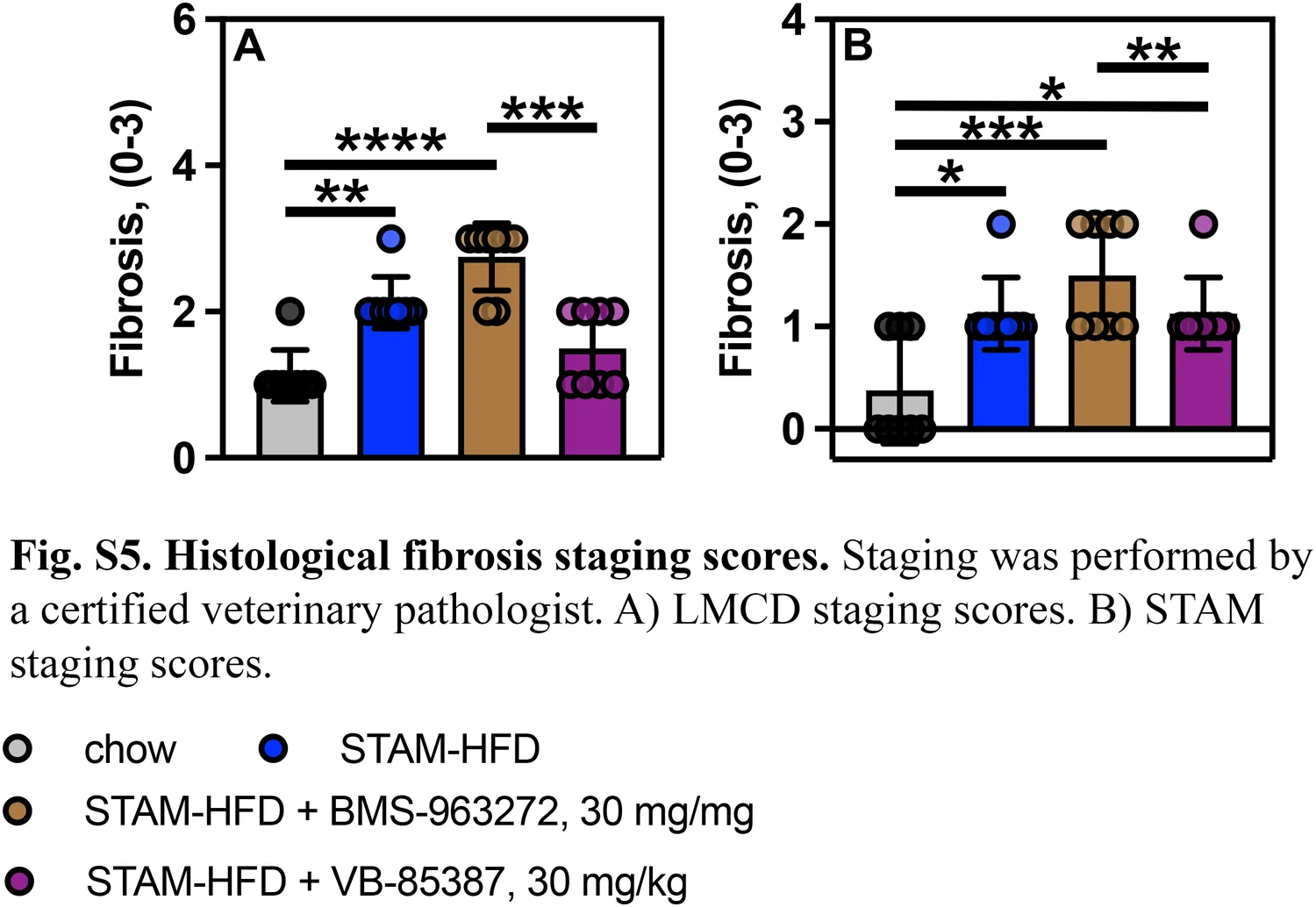


**Fig. S6:**
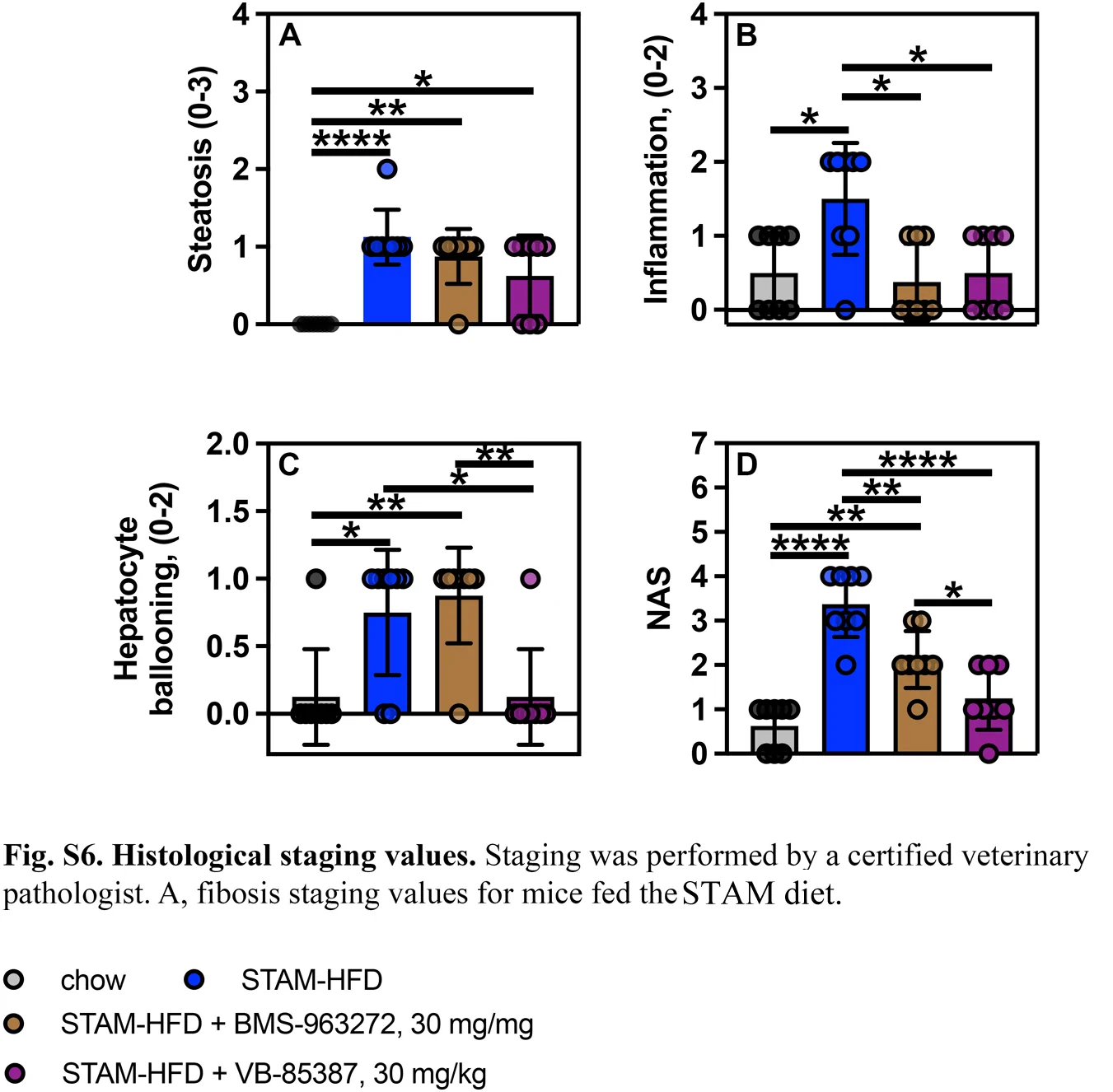


**Fig. S7:**
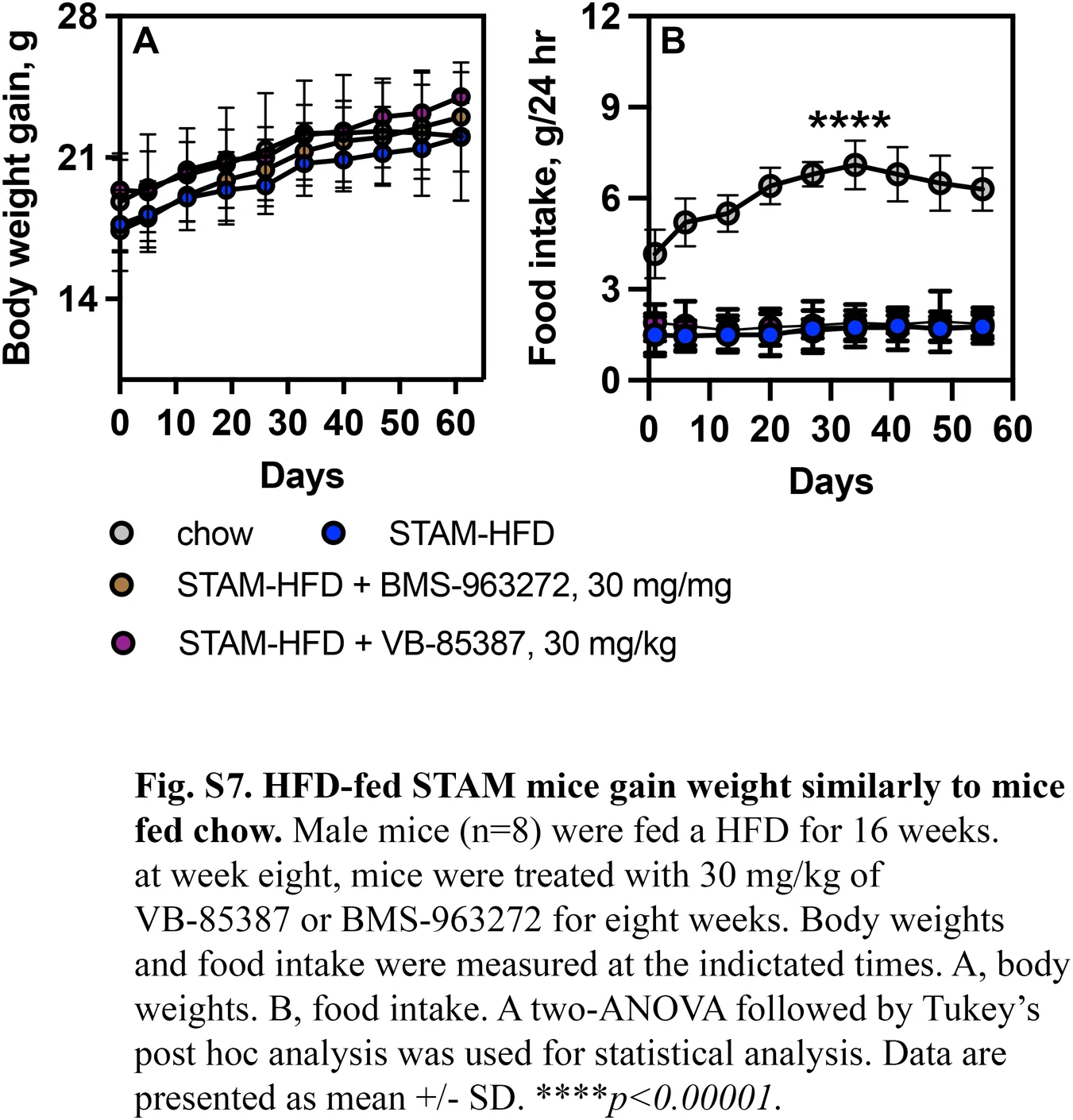


**Fig. S8:**
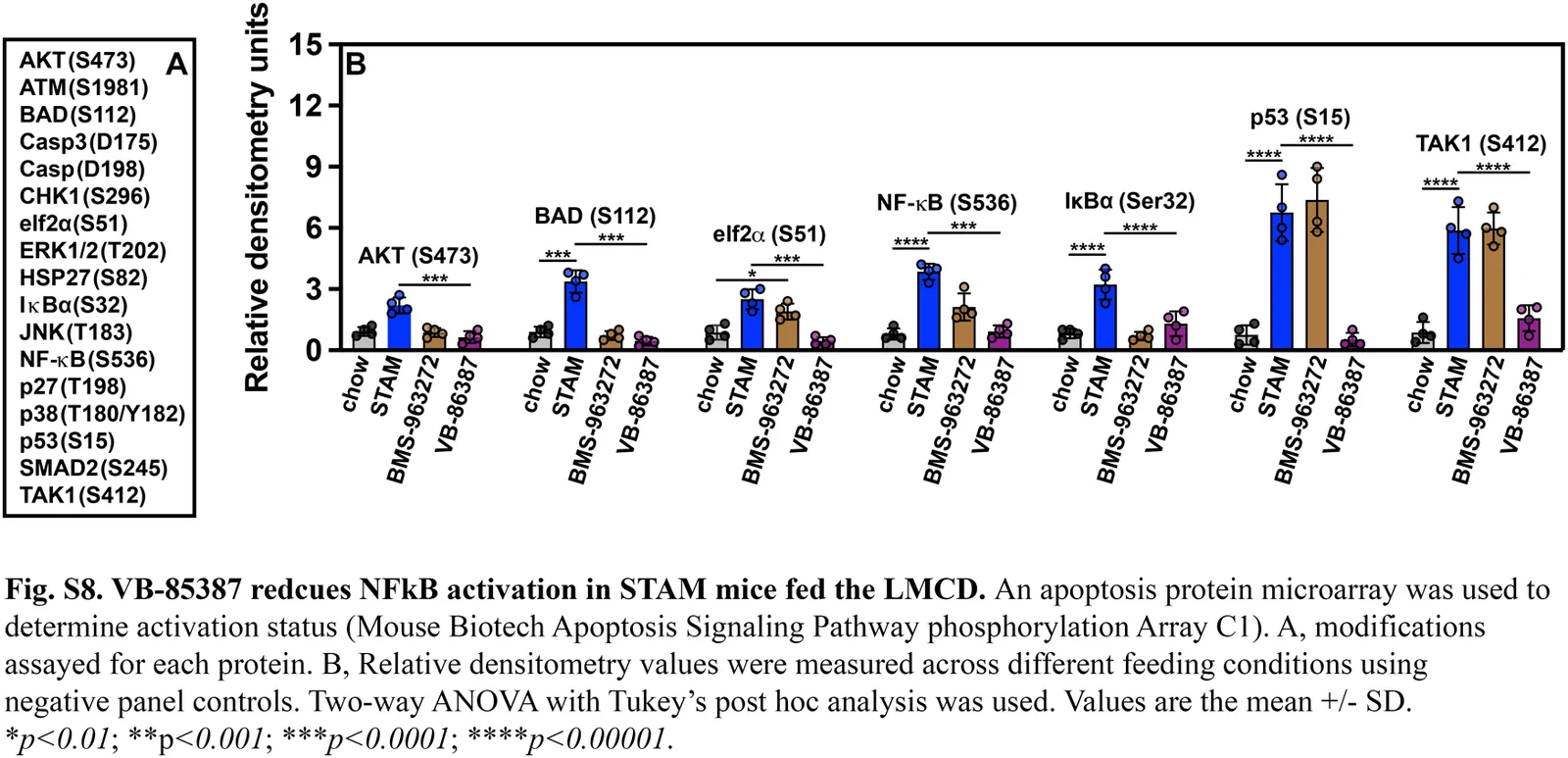


**Fig. S9:**
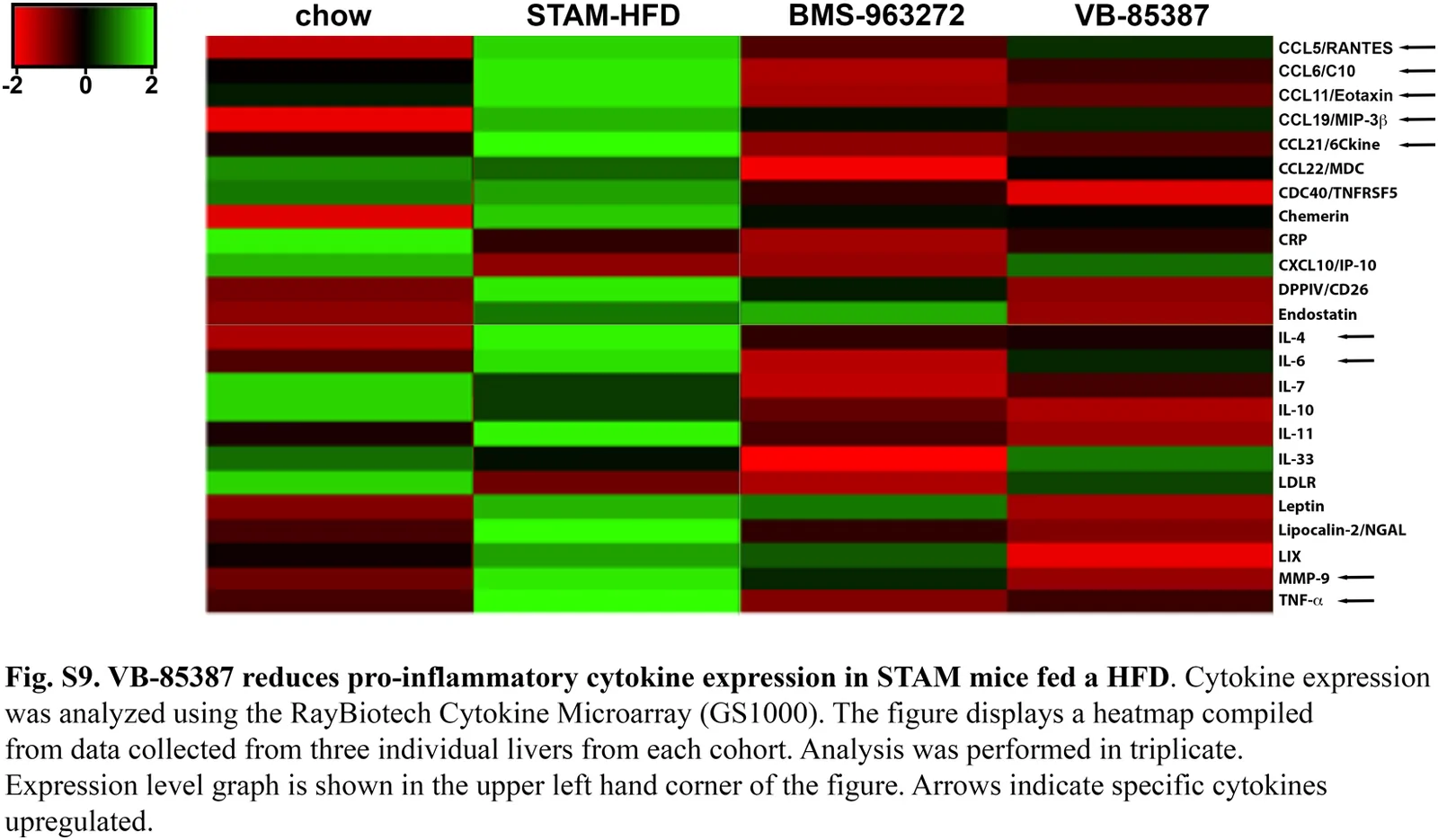


**Fig. S10:**