# Supplementary material for: The monoacylglycerol acyltransferase 2 inhibitor VB-85387 reduces MASLD
Source: J Lipid Res. 2026 Sep 2;67(10):101137. doi: 10.1016/j.jlr.2026.101137 (PMC13639739; doi:10.1016/j.jlr.2026.101137)
Supplement: Supplementary file 1 [file mmc1.docx]

Table S1. Liver enzyme levels in LMCD and STAM mice

| LMCD | | | | |
| --- | --- | --- | --- | --- |
| Enzyme | chow | LMCD | LMCD + BMS-963272 | LMCD + VB-85387 |
| ALT, IU | 38.25 ± 24.53 | 223.8 ± 23.83 | 153.2 ± 16.15^a^ | 142.00 ± 12.58^a^ |
| AST, IU | 64.12 + 14.57 | 267.7 + 26.71 | 251 + 22.86 | 186.50 + 17.63^b^ |
|  |  |  |  |  |
| STAM-HFD | | | | |
| Enzyme | chow | STAM-HFD | STAM-HFD + BMS-963272 | STAM-HFD + VB-85387 |
| ALT, IU | 95.32 ± 20.54 | 179.25 ± 33.19 | 91.75 ± 24.85^a^ | 65.37 ± 25.98^a^ |
| AST, IU | 99.14 ± 28.62 | 241.23 ± 41.86 | 124.62 ± 27.84^c^ | 105.37 ± 22.85^b^ |

n=8

^a^*p≤0.0001*

^b^*p≤0.001*

^c^*p≤0.01*

*compared to mice fed the LMC*
