# Supplementary material for: The monoacylglycerol acyltransferase 2 inhibitor VB-85387 reduces MASLD
Source: J Lipid Res. 2026 Sep 2;67(10):101137. doi: 10.1016/j.jlr.2026.101137 (PMC13639739; doi:10.1016/j.jlr.2026.101137)
Supplement: Supplementary file 2 [file mmc2.docx]

Table S1. Table S2. Hydroxyproline levels in LMCD and STAM mice

| LMCD | | | | |
| --- | --- | --- | --- | --- |
| Enzyme | chow | LMCD | LMCD + BMS-963272 | LMCD + VB-85387 |
| Hydroxyproline, μg/g tissue | 195.25 + 22.25 | 1346 + 62.82^a^ | 1337 + 61.60^a^ | 900.5 + 54.14^b^ |
|  |  |  |  |  |
| STAM-HFD | | | | |
| Enzyme | chow | STAM-HFD | STAM-HFD + BMS-963272 | STAM-HFD + VB-85387 |
| Hydroxyproline, μg/g tissue | 255.00 + 29.75 | 595.62 + 32.10^a^ | 420.00 + 30.30^b^ | 267.50 + 27.144 |
|  | | | | |

n=8

^a^*p≤0.0001*

^b^*p≤0.01*

*compared to mice fed chow*
